# A stapled chromogranin A-derived peptide homes in on tumors that express αvβ6 or αvβ8 integrins

**DOI:** 10.7150/ijbs.76148

**Published:** 2023-01-01

**Authors:** Matteo Monieri, Paolo Rainone, Angelina Sacchi, Alessandro Gori, Anna Maria Gasparri, Angela Coliva, Antonio Citro, Benedetta Ferrara, Martina Policardi, Silvia Valtorta, Arianna Pocaterra, Massimo Alfano, Dean Sheppard, Lorenzo Piemonti, Rosa Maria Moresco, Angelo Corti, Flavio Curnis

**Affiliations:** 1 Division of Experimental Oncology, IRCCS San Raffaele Scientific Institute, Milan, Italy.; 2 Department of Medicine and Surgery, University of Milano-Bicocca, Monza, Italy.; 3 Experimental Imaging Center, IRCCS San Raffaele Scientific Institute, Milan, Italy.; 4 Istituto di Scienze e Tecnologie Chimiche, C.N.R., Milan, Italy.; 5 Diabetes Research Institute, IRCCS San Raffaele Scientific Institute, Milan, Italy.; 6 Division of Immunology Transplantation and Infectious Diseases, IRCCS San Raffaele Scientific Institute, Milan, Italy.; 7 Lung Biology Center, Department of Medicine, University of California, San Francisco, San Francisco, CA, USA.; 8 Institute of Molecular Bioimaging and Physiology of C.N.R., Segrate, Italy.; 9 Faculty of Medicine and Surgery, Vita-Salute San Raffaele University, Milan, Italy.

**Keywords:** RGD motif, αvβ6 and αvβ8 integrins, TGFβ, chromogranin A, cancer.

## Abstract

**Rationale**: The αvβ6- and αvβ8-integrins, two cell-adhesion receptors upregulated in many tumors and involved in the activation of the latency associated peptide (LAP)/TGFβ complex, represent potential targets for tumor imaging and therapy. We investigated the tumor-homing properties of a chromogranin A-derived peptide containing an RGDL motif followed by a chemically stapled alpha-helix (called “**5a”**), which selectively recognizes the LAP/TGFβ complex-binding site of αvβ6 and αvβ8.

**Methods:** Peptide **5a** was labeled with IRDye 800CW (a near-infrared fluorescent dye) or with ^18^F-NOTA (a label for positron emission tomography (PET)); the integrin-binding properties of free peptide and conjugates were then investigated using purified αvβ6/αvβ8 integrins and various αvβ6/αvβ8 single - or double-positive cancer cells; tumor-homing, biodistribution and imaging properties of the conjugates were investigated in subcutaneous and orthotopic αvβ6-positive carcinomas of the pancreas, and in mice bearing subcutaneous αvβ8-positive prostate tumors.

**Results:** *In vitro* studies showed that **5a** can bind both integrins with high affinity and inhibits cell-mediated TGFβ activation. The **5a-**IRDye and **5a**-NOTA conjugates could bind purified αvβ6/αvβ8 integrins with no loss of affinity compared to free peptide, and selectively recognized various αvβ6/αvβ8 single- or double-positive cancer cells, including cells from pancreatic carcinoma, melanoma, oral mucosa, bladder and prostate cancer. *In vivo* static and dynamic optical near-infrared and PET/CT imaging and biodistribution studies, performed in mice with subcutaneous and orthotopic αvβ6-positive carcinomas of the pancreas, showed high target-specific uptake of fluorescence- and radio-labeled peptide by tumors and low non-specific uptake in other organs and tissues, except for excretory organs. Significant target-specific uptake of fluorescence-labeled peptide was also observed in mice bearing αvβ8-positive prostate tumors.

**Conclusions:** The results indicate that **5a** can home to αvβ6- and/or αvβ8-positive tumors, suggesting that this peptide can be exploited as a ligand for delivering imaging or anticancer agents to αvβ6/αvβ8 single- or double-positive tumors, or as a tumor-homing inhibitor of these TGFβ activators.

## Introduction

Integrin αvβ6 is an epithelial-specific cell-surface receptor poorly expressed in normal adult tissues and highly expressed during wound healing, tissue remodeling, and embryogenesis 1, 2. This integrin is also overexpressed by several types of cancer cells, such as head and neck squamous cell carcinoma, pancreatic ductal adenocarcinoma (PDAC), breast, liver, colon, and ovarian cancers, and others 1-7; αvβ6 expression level is a prognostic indicator of poor survival in patients with various types of tumors 3, 6, 8-10. The integrin αvβ8 is another cell-surface receptor expressed by astrocytes, dendritic cells, mural mesangial cells, and by various carcinoma cells and tumor infiltrating regulatory T cells 11-13; enhanced αvβ8 expression mediates radio/chemo-resistance in pancreatic cancer 13 and serves as a marker of poor prognosis in colon carcinoma patients 14.

Considering the enhanced expression of these integrins by several types of cancer cells, compounds that target these integrins in tumors are of great experimental and clinical interest, as they could be used either for delivering imaging and therapeutic agents to tumors, or to modulate their activity.

According to this view, various ligands of αvβ6 have been developed, such as the foot-and-mouth disease virus-derived peptide A20FMDV2, the cyclic peptide c[RGDLATK] (Cycratide), the trimerized nonapeptide Trivehexin, the cysteine knot peptides, and the sunflower trypsin inhibitor-derived peptides 15. These compounds, coupled to tumor imaging agents, are currently being tested in cancer patients 16-20. Similarly, a radiolabeled trimerized peptide c[GLRGDLp(*N*Me)K], an αvβ8 ligand, has shown encouraging preclinical imaging results in a murine αvβ8-positive melanoma model 21. Furthermore, considering that both αvβ6 and αvβ8 can activate TGFβ (a potent immunosuppressive cytokine) by interacting with the RGD sequence of the inactive latency-associated peptide (LAP)-TGFβ complex 1, 7, 11, compounds capable of targeting and blocking this integrin may have therapeutic activity 22. This view is supported by the results of recent studies showing that anti-αvβ8 antibodies can inhibit TGFβ activation in murine tumors and elicit durable antitumor immunity 12, 23, while anti-αvβ6 antibodies can inhibit the growth of αvβ6-positive tumors, again through a TGFβ-regulated mechanism 24.

Thus, the development of bi-specific tumor-homing compounds that target the active site of both αvβ6 and αvβ8 integrins in tumors may represent an important advance in this field. With this in mind, we have investigated the tumor-homing properties of a peptide that selectively binds the active site of both αvβ6 and αvβ8 with high affinity 25. This peptide, derived from the region 39-63 of human chromogranin A (a neurosecretory protein), contains an RGDL motif, followed by an alpha-helix chemically stapled with a triazole bridge, which binds the RGD binding site of αvβ6 with interactions similar to those observed with the proTGFβ1/αvβ6 complex 25, 26. To assess its capability to home in on αvβ6- and/or αvβ8-positive tumors we have labeled this peptide with optical- and radio-imaging compounds, analyzed their capability of recognizing various αvβ6/αvβ8 double- or single-positive cancer cells, and investigated their tumor-homing and biodistribution properties in subcutaneous and orthotopic murine models of pancreatic cancer (αvβ6^+^ and αvβ8^-^) and prostate cancer (αvβ6^-^ and αvβ8^+^). We show that this peptide binds αvβ6/αvβ8 single- or double-positive cancer cells *in vitro* and efficiently accumulates in αvβ6- or αvβ8-positive tumors through a receptor-mediated mechanism. Furthermore, we show that this peptide can inhibit TGFβ activation by cancer cells, suggesting that this compound is an inhibitor of αvβ6 and αvβ8 endowed of tumor-homing properties.

## Materials and methods

Full details of all methods, reagents and equipment used are presented in the Supplemental Materials.

### Peptide synthesis, purification, and characterization

Peptides were prepared by chemical synthesis; their identity and purity were checked by mass spectrometry (MS) and reverse-phase HPLC analysis. The affinity of peptides for αvβ6-coated plates was determined by competitive binding assay using an isoDGR peptide (an RGD mimetic) labeled with peroxidase as a probe for the integrin-binding site 25. Cell adhesion assays were carried out as described previously 26.

### TGFβ bioassay

Quantification of bioactive TGFβ in TRAMP-C2 cell supernatant was carried using TGFβ-Reporter HEK-Blue™ cells (InvivoGen) according to the supplier's recommendations.

### Conjugation of peptides to maleimide-IRDye 800CW or maleimide-NOTA

Peptides with a N-terminal cysteine were coupled, via a thiol group, to maleimide-IRDye®800CW (IRDye, LI-COR) or 1,4,7-triazacyclononane-1,4-bis-acetic acid-7-maleimidoethylacetamide (maleimide-NOTA, CheMatech). The conjugates were purified by RP-HPLC; their identity and purity were confirmed by MS and HPLC analysis.

### Binding of peptide-IRDye conjugates to αvβ6 and αvβ8 and to cultured integrin-expressing cells

The affinity of peptide-IRDye conjugates for αvβ6 and αvβ8 integrins was determined by a direct binding assay using microtiter plates coated with αvβ6 or αvβ8 (Bio-Techne). After 1 h of incubation, the plates were washed; bound fluorescence was quantified by scanning the plates with an Odyssey CLx near-infrared fluorescence imaging system (LI-COR).

To assess the binding of peptide-IRDye conjugates to cells various amounts of conjugates (range 0.32-200 nM) were added to cultured cells and left to incubate for 1 h at 37 °C. After three washings, the cells were fixed and analyzed by scanning the plate with the Odyssey CLx.

### *In vivo* studies in animal models

Procedures involving laboratory mice and their care were approved by the Ospedale San Raffaele Animal Care and Use Committee and approved by the Minister of Health. The study was performed at the San Raffaele Hospital (authorized organization) according to institutional guidelines and in compliance with national and international laws and guidelines.

The tumor-homing properties of the labeled peptides were evaluated using a) subcutaneous mouse tumor models of pancreatic and prostate cancer, and b) an orthotopic mouse model of pancreatic cancer (*see* Supplemental Materials).

### Near-infrared imaging studies

Mice were injected with peptide-IRDye conjugate (~1 nmol) into the tail vein and imaged at various time points (0-72 h) using the IVIS Spectrum CT Imaging System (PerkinElmer) (*see* Supplemental Materials). For blocking experiments, mice were injected intravenously with unlabeled peptide **5a** (128 nmol/mouse) 10 min before **5a**-IRDye.

### PET imaging and biodistribution studies

The **5a**-NOTA conjugate was radiolabeled with ^18^F using a modified Tracerlab FX-N automatic module (GE Healthcare). Full details are presented in the Supplemental Materials. The radiotracer uptake (~4 MBq/mice, in 100 μl of water containing <10% ethanol) was monitored by whole-body PET/CT using the preclinical β-cube® and X-cube® scanners (Molecubes), respectively. For *ex vivo* biodistribution, mice were euthanized, and tumor and selected organs were collected, rinsed, weighed, and analyzed for their radioactivity content using a γ-counter (LKB Compugamma CS 1282).

## Results

### Peptide 5a binds αvβ6 and αvβ8 with high affinity and inhibits cell-mediated TGFβ activation

We previously developed a chromogranin A-derived peptide (FETLRGDLRILSILR**X_1_**QNL**X_2_**KELQD, peptide **5**), capable of recognizing αvβ6 and αvβ8 with high affinity and selectivity (*Ki*=0.6 nM and 3.2 nM, respectively) 25. This peptide contains the RGDLXXL integrin recognition motif followed by an amphipathic alpha-helix chemically stabilized by a triazole bridge between the propargylglycine (**X_1_**) and azidolysine (**X_2_**) residues.

**Figure 1:**
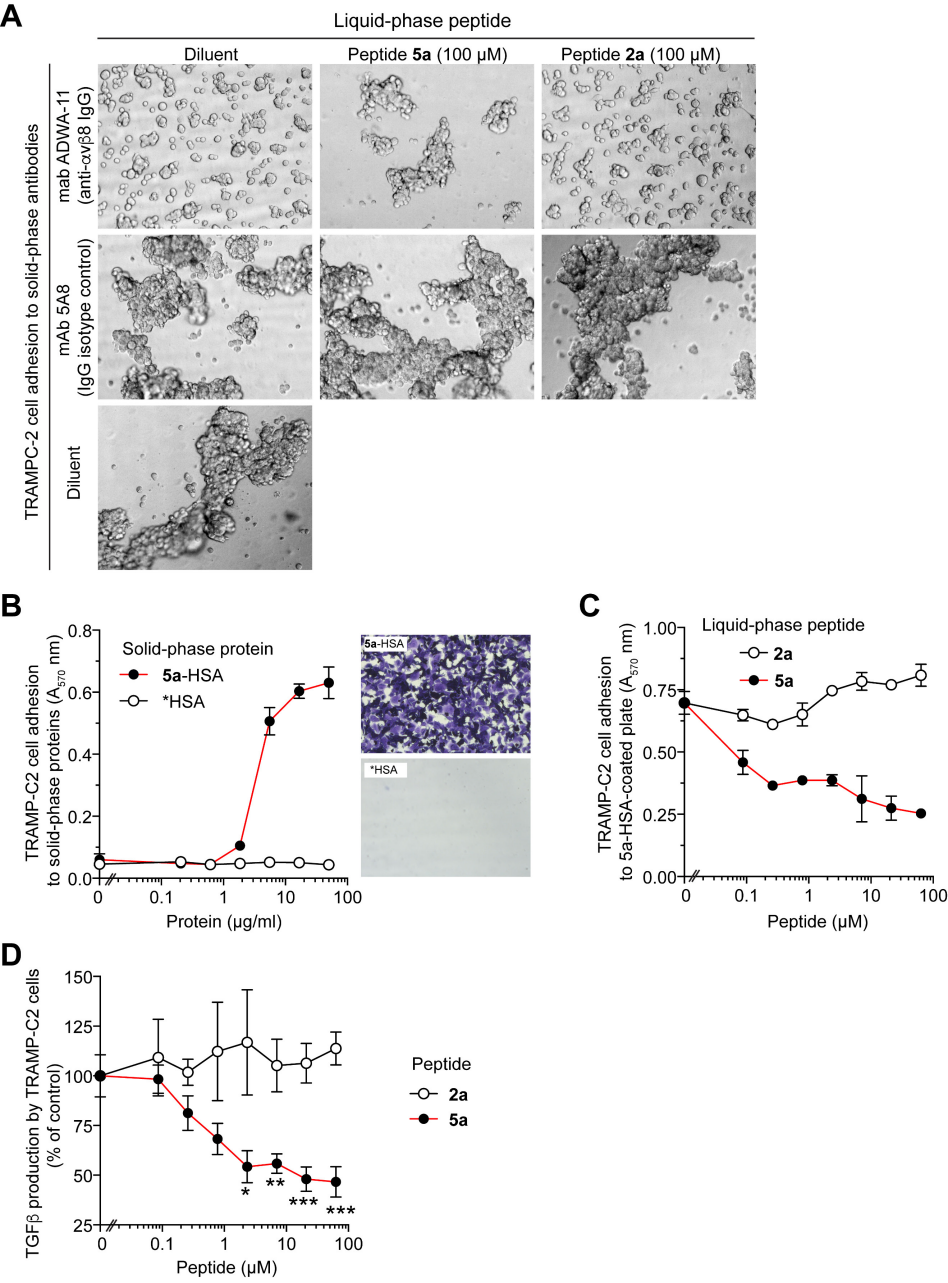
** Peptide 5a prevents the adhesion of TRAMP-C2 cells (αvβ8-positive) to plates coated with an anti-αvβ8 antibody or with a peptide 5a-albumin conjugate and inhibits cell-mediated TGFβ activation. (A)** Effect of 5a and 2a on the adhesion of TRAMPC-2 cells to PVC-microplates coated with anti-αvβ8 mAb ADWA-11 or mAb 5A8 (a negative control). Cells were mixed with or without the peptides (100 µM) and analyzed by bright-field microscopy after 2h. Representative photographs (10x magnification) are shown. **(B)** Adhesion of TRAMP-C2 cells to PVC-microplates coated with a 5a-human serum albumin conjugate (5a-HSA), or with a control conjugate made without peptide (*HSA), after 2 h of incubation. Non-adherent cells were removed; adherent cells were stained with crystal violet and quantified by spectrophotometric analysis (A_570_nm) (mean±SE, n=3). Representative photomicrographs of wells coated with 5a-HSA or *HSA (16.6 µg/ml) are shown. **(C)** Effect of 5a and 2a on the adhesion of TRAMPC-2 cells to PVC-microplates coated with 5a-HSA (10 µg/ml). Adherent cells were stained with crystal violet and analyzed spectrophotometrically (mean±SE, n=2-3). **(D)** Effect of 5a and 2a on TGFβ activation by TRAMP-C2 cells. Cells were seeded on cell culture microplates, left to adhere for 3 h and treated with 5a or 2a for 16 h. The amount of active TGFβ in the supernatant was quantified using a bioassay based on HEK-Blue™ TGFβ cells. Cumulative results of two independent experiments are shown (mean±SE, n=4-6 wells). *, P<0.05; **, P<0.01; ***P <0.001 by *two-tail t-test.*

To couple peptide **5** to imaging compounds containing maleimide groups, we fused a cysteine residue to its N-terminus (peptide** 5a**, *see* **Figure S1**). The capability of peptide **5**, **5a** and **2a** (the latter containing RGE instead of RGD)** (Table 1)** to inhibit the interaction of αvβ6 with an isoDGR-peroxidase conjugate (a probe for the RGD-binding site of integrins 25) was then analyzed. Peptide **5** and **5a,** but not** 2a,** could bind αvβ6 with similar affinities **(Table 1 and Figure S2),** pointing to a crucial role of RGD for αvβ6 recognition. Peptide **5a**, but not **2a**, inhibited the adhesion of TRAMP-C2 prostate cancer cells (αvβ6^-^ and αvβ8^+^) to a neutralizing anti-αvβ8 antibody **(Fig. 1A)**, whereas it promoted cell-adhesion when adsorbed onto the solid-phase **(Figure 1B and C)**; furthermore **5a**, but not **2a**, inhibited the capability of these cells to activate TGFβ **(Figure 1D)**. Overall, these results suggest that a) the cysteine residue added to peptide **5** does not impair its ability to bind αvβ6 and αvβ8 and b) peptide **5a** can inhibit cell-mediated TGFβ activation.

### The 5a-IRDye conjugate binds recombinant human and murine αvβ6 and αvβ8

Peptide **5a, 2a,** and cysteine (**Cys**) were then coupled to maleimide-IRDye 800CW (IRDye), a near-infrared dye, to generate the **5a**-,** 2a**-**, Cys-**IRDye conjugates (*see* **Table S1, S2** and **Figure S3** for product characterization). Direct integrin-binding assays showed that **5a**-IRDye, but not **2a**-IRDye or **Cys**-IRDye, could bind microtiter plates coated with human and murine αvβ6 or αvβ8 with affinity in the low nanomolar range **(Figure S4).** Thus, the IRDye moiety does not impair the αvβ6 and αvβ8 binding properties of** 5a**.

### 5a-IRDye binds to αvβ6/αvβ8 single- or double-positive cancer cells

To evaluate the ability of **5a**-IRDye to recognize αvβ6 on the cell surface, we then analyzed the interaction of **5a-**, **2a-** or **Cys**-IRDye with αvβ6-positive and -negative cells, including human BxPC-3 cells (αvβ6^+^/αvβ8^-^), murine 5M7101 pancreatic adenocarcinoma cells (αvβ6^+^ and αvβ8^-^), and human umbilical vein endothelial cells (HUVECs) (αvβ6^-^ and αvβ8^-^) **(Figure S5A and S6A)**. As expected,** 5a**-IRDye could bind BxPC-3 and 5M7101 cells, but not HUVECs, while little or no binding of **2a**-IRDye and **Cys-**IRDye (control conjugates) to all cells tested was observed **(Figure S5B and S6B)**. Binding of **5a**-IRDye to BxPC-3 cells was inhibited by the αvβ6-blocking antibody 10D5 (but not by an isotype-matched control antibody), and by an excess of **5a** or A20FMDV2 (a known ligand of αvβ6), but not by **2a** (**Figure S5C** and** D**). These results confirm the hypothesis that **5a**-IRDye binding to cells is mediated αvβ6 and that its RGD sequence is critical for binding.

 The ability of **5a**-IRDye to recognize αvβ8^+^ and αvβ6^+^/αvβ8^+^ cells was investigated using human MeWo melanoma cells (αvβ6^-^/αvβ8^+^), murine TRAMP-C2 prostate cancer cells (αvβ6^-^/αvβ8^+^), human 5637 bladder cancer cells (αvβ6^+^/αvβ8^+^) and human BHY oral squamous carcinoma cells (αvβ6^+^/αvβ8^low^)** (Figure S7A)**. As expected, **5a**-IRDye could bind these cells in a dose-dependent manner and more efficiently than the control conjugates **(Figure S7B),** indicating that this peptide can recognize human and murine αvβ8 also when expressed on the cell surface.

### 5a-IRDye homes in on subcutaneous αvβ6^+^-pancreatic adenocarcinomas

The ability of **5a**-IRDye to bind αvβ6 *in vivo* was assessed using a subcutaneous xenograft model of pancreatic adenocarcinoma based on human BxPC-3 αvβ6^+^/αvβ8^-^ cells implanted in NGS, and a subcutaneous syngeneic model based on murine 5M7101 cells implanted in C57BL/6N mice. *In vivo* NIR-fluorescence analysis of BxPC-3 tumors showed maximal **5a**-IRDye uptake 1 h after injection, with a tumor-to-background ratio (TBR) of about 7; significant signal was still visible 24 h later (TBR: ~4) **(Figure S8A and B)**. *Ex vivo* analysis of tumor, pancreas, and kidney showed that the uptake was higher in tumor and kidney than in **pancreas (Figure S8C)**. Of note, the tumor-to-pancreas ratio was ~12, indicating that **5a**-IRDye accumulated better in pancreatic tumors than in normal pancreas.

A TBR of ~8 was observed in the 5M7101 model at 24 h **(Figure S8D)**. These results suggest that **5a**-IRDye can home to human and murine αvβ6^+^ pancreatic adenocarcinomas.

**Figure 2:**
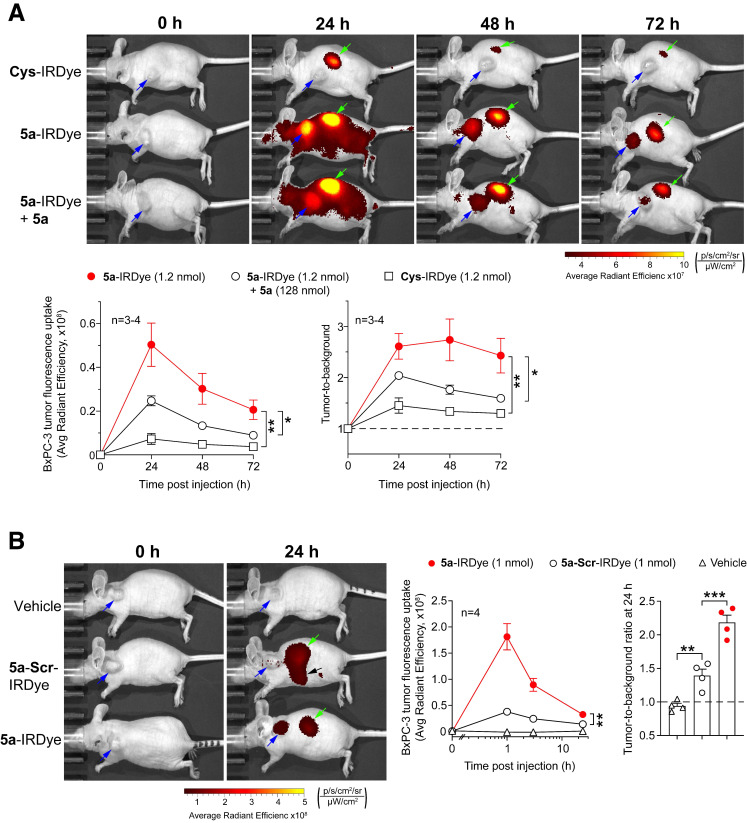
** The uptake of 5a-IRDye by subcutaneous BxPC-3 tumor depends on the 5a moiety. (A)** Nude mice bearing-BxPC-3 tumors were injected i.v. with the indicated amount of **Cys-**IRDye, **5a**-IRDye, or **5a**-IRDye plus an excess of **5a,** and imaged at the indicated time points using an IVIS imaging system, 30-35 days after tumor implantation. Pseudocolor images of representative mice for each group of treatment are shown (*blue* and *green* arrows indicate tumor and kidney, respectively). Quantification of the fluorescence uptake by BxPC-3 tumors and tumor-to-background ratio are shown in the lower panels (graphs, means ± SE of 3-4 mice/group; *, P<0.05 and **, P<0.01 by *ordinary one-way ANOVA* of the area under the curve for each tumor using GraphPad Prism software). **(B)** Similar experiment showing the tumor uptake of **5a**-IRDye compared to **5a**-**Scr**-IRDye. *Blue* and *green* arrows in pseudocolor images indicate tumor and kidney, respectively, while *black arrow* indicates the intestine. **, P<0.01; ***P<0.001 by *ordinary one-way ANOVA*. *Black* arrow indicates **5a**-**Scr**-IRDye accumulation in the intestine (as confirmed by *ex vivo* analysis of the isolated organ, not shown).

To verify the specificity of **5a**-IRDye, we compared the uptake of this conjugate and two controls made with Cys or a scrambled sequence in place of **5a** (**Cys-**IRDye **5a**-**Scr**-IRDye, *see*** Table 1**) by BxPC-3 tumors implanted in nude mice, a hairless model. As expected, **5a**-IRDye accumulated in tumors more efficiently than **Cys-**IRDye and **5a**-**Scr**-IRDye **(Figure 2A and B)**. The uptake of **5a**-IRDye was significantly inhibited by prior administration of an excess of **5a (Figure 2B)**, suggesting it was mediated by the **5a** moiety.

### 5a-IRDye homes in on orthotopic αvβ6^+^-pancreatic adenocarcinomas (BxPC-3)

**Figure 3:**
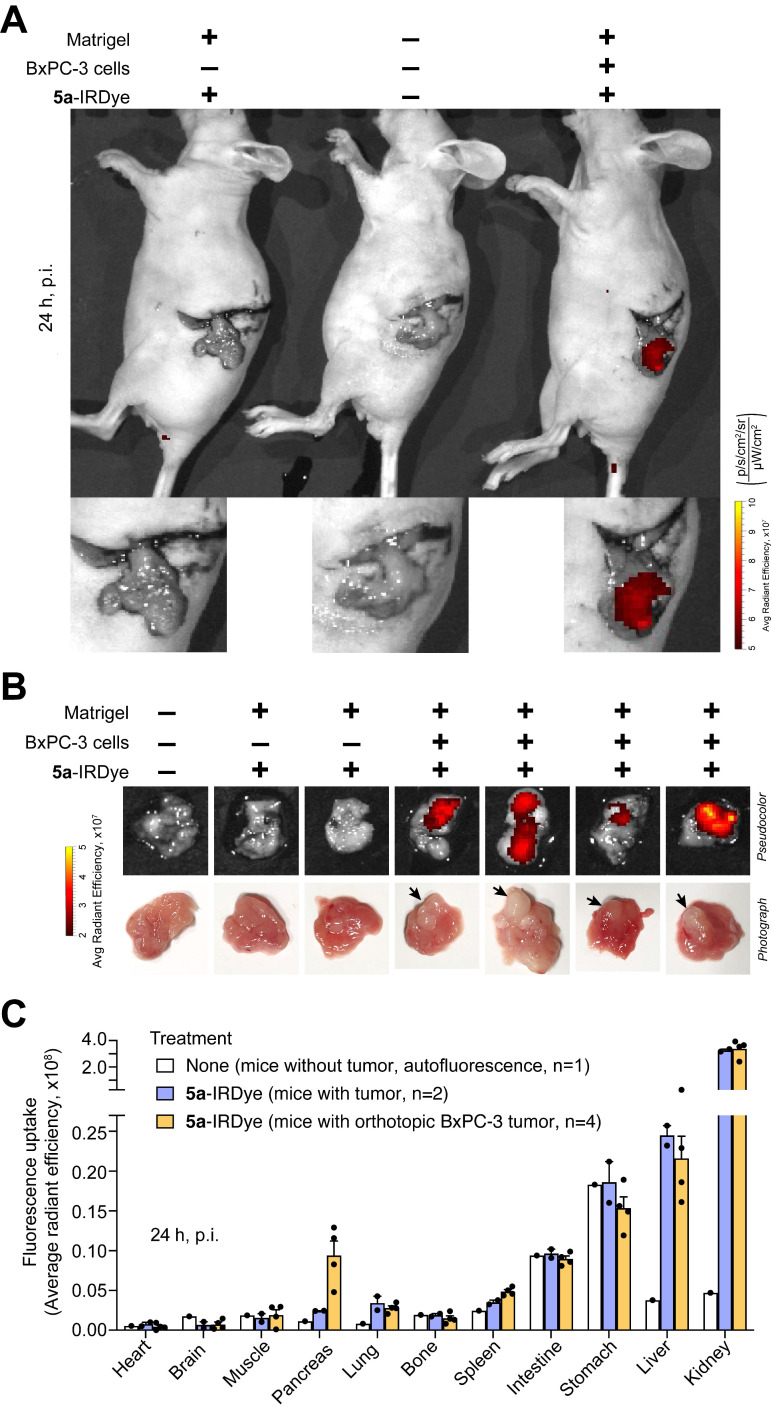
** 5a-IRDye homes in on orthotopically implanted BxPC-3 tumors.** Nu/nu mice were orthotopically implanted with a Matrigel solution containing BxPC-3 cells or a Matrigel solution without cells (n=3-4 mice). After 18 days, mice were injected i.v. with **5a**-IRDye (1.2 nmol), and NIR images were acquired after 24 h. A control mouse, which was not surgically manipulated, was injected with the vehicle and used as a reference for quantification of autofluorescence in the NIR region. **(A)** Representative fluorescence images of the exposed pancreas of control and tumor-bearing mice injected with or without **5a**-IRDye as indicated. **(B)** *Ex vivo* fluorescence imaging and photographs of the removed pancreas. *Arrow*, pancreatic cancer lesions. **(C)** *Ex vivo* biodistribution of **5a**-IRDye in selected organs. *Bars*: mean ± SE of 1-4 mice.

The ability of **5a**-IRDye to home in on pancreatic tumors was also investigated in an orthotopic model of pancreatic adenocarcinoma based on surgical implantation of BxPC-3 cells, suspended in Matrigel, into the head of the pancreas of nude mice. Mice implanted with Matrigel (without cells) served as controls. NIR fluorescence analysis of the surgically exposed pancreas of mice, 24 h after administration, showed higher accumulation of **5a**-IRDye in the pancreas of mice with BxPC-3 tumors than in the pancreas of control mice **(Figure 3A)**. NIR fluorescence analysis of the excised pancreas showed that the ratio of tumor-to-normal pancreas was ~4 **(Figure 3B, C and Figure S9)**. Noteworthy, larger tumor lesions accumulated more dye than small lesions **(Figure 3B)**.

Analysis of other explanted organs showed that **5a**-IRDye accumulated also in kidney, lung, and liver, and little or not at all in heart, brain, muscle, bone, spleen, intestine, and stomach **(Figure 3C and Figure S9)**. The higher accumulation of **5a**-IRDye in kidney and urine (not shown), compared with the other organs, is likely related to renal clearance.

### PET imaging of subcutaneous αvβ6^+^-pancreatic adenocarcinomas (BxPC-3) with 5a-NOTA-^18^F

To further evaluate the ability of **5a** to recognize αvβ6 *in vivo* and to home in on αvβ6-positive tumors, we coupled this peptide with maleimide-NOTA (**5a**-NOTA) to allow radiolabeling with ^18^F **(Table S1, S2 and Scheme S2)**. The **2a**-NOTA conjugate was also prepared (negative control). RP-HPLC and MS analysis of the conjugates showed that they were homogeneous and with the expected molecular weight **(Figure S10A and B)**.

Competitive αvβ6-binding assays showed that **5a**-NOTA, but not **2a**-NOTA, binds αvβ6 with an affinity similar to that of **5a**, indicating that the NOTA moiety does not interfere with αvβ6 recognition **(Figure S10C)**.

**5a**-NOTA, labeled with ^18^F (**5a-**NOTA-^18^F**)**, showed radiochemical purity >96%, specific activity of 2.2-13.9 MBq/nmol, and good stability after 4 h storage at room temperature **(Figure S10D)**.

**Figure 4:**
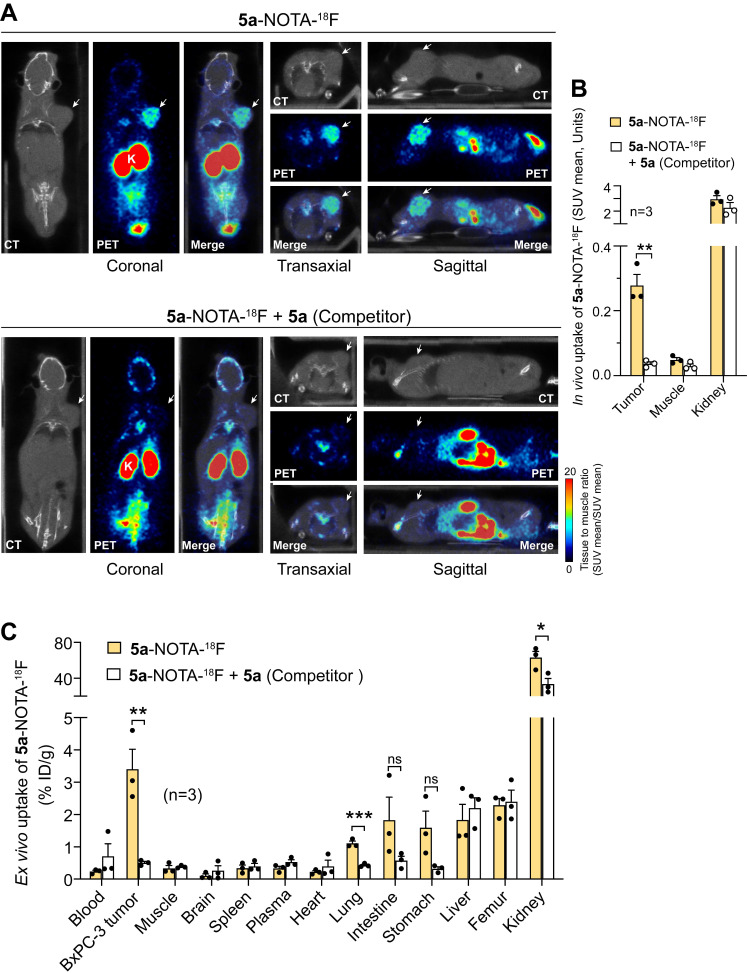
** Competition of 5a-NOTA-^18^F uptake by unlabeled peptide 5a in the subcutaneous BxPC-3 tumor model.** BxPC-3 tumors-bearing NGS mice were injected i.v. with or without unlabeled 5a (130 nmol/mouse, *Competitor*) and 10 min later, with 5a-NOTA-^18^F (~ 3 MBq/animal, i.v.). Radiotracer uptake was assessed after 2 h by whole-body PET/CT **(A, B)** or with a gamma-counter for biodistribution studies **(C)**. *Bars*, mean ± SE (n=3 mice). *Arrow*, BxPC-3 tumor. *, P<0.05, **, P<0.01 and ***P<0.001 by *two-tail t-test*.

Whole-body PET/CT scan of mice bearing subcutaneous BxPC-3 tumors, performed 1, 2, and 4 h after **5a-**NOTA-^18^F administration, showed radiotracer accumulation in tumors and kidneys, but not in muscles or femurs, i.e., tissues that do not express αvβ6 **(Figure S11A)**. Notably, the high and progressive retention of the radiotracer in tumors compared to muscles, but not or much less in femurs, suggest a specific mechanism of uptake **(Figure S11B)**. Accordingly, tumor uptake of **5a-**NOTA-^18^F, 2 h post-injection, was almost completely inhibited by prior administration of an excess of **5a (Figure 4A and B)**, pointing to a specific mechanism involving ligand-receptor interactions.

*Ex vivo* biodistribution data (obtained 2 h post-injection) confirmed that the radiotracer accumulates in tumors in a specific manner, as shown by the marked decrease in tumor uptake (from 3.5% to less than 0.5% of the injected dose (ID)/g of tissue) in mice pretreated with an excess of **5a (Figure 4C)**. Lower, albeit specific, accumulation of **5a-**NOTA-^18^F was also observed in lung (1.2% ID/g). Uptake in brain, heart, spleen, blood, and muscle was less than 0.5% ID/g and was not displaced by the free peptide. In addition, some accumulation (about 2% of ID/g) was also observed in intestine, femur, liver, and stomach. In this case, however, no significant reduction was caused by unlabeled **5a**, arguing against a peptide-mediated mechanism of accumulation in these organs. Finally, high radiotracer levels were also observed in the kidneys (about 80% ID/g), probably due to renal clearance of the conjugate.

### 5a-IRDye accumulates on αvβ8-positive prostate tumors (TRAMP-C2)

Finally, the ability of **5a** to home to αvβ8-positive tumors was investigated in the TRAMP-C2 prostate tumor model (αvβ6^-^/αvβ8^+^), implanted subcutaneously in immunodeficient mice. Uptake of **5a**-IRDye by these tumors was significantly inhibited by an excess of **5a (Figure S12)**, suggesting a **5a-**mediated mechanism of uptake. The low, albeit specific, uptake of **5a**-IRDye in this model compared with αvβ6-positive pancreatic adenocarcinomas, is likely related to the lower αvβ8-expression of TRAMP-C2 cells compared with that of BxPC-3 cells, as observed by flow-cytometry analysis **(Figure S5A and Figure S7A)**. Nevertheless, these results lend further support to the hypothesis that **5a** can recognize αvβ8 *in vivo.*

## Discussion

This work demonstrates that **5a**, a chromogranin A-derived peptide with high affinity for human αvβ6 and αvβ8, homes in on αvβ6 or αvβ8 integrin-positive tumors. In these experimental setups, **5a** was coupled *via* cysteine to maleimide-IRDye® 800CW (a NIR fluorescent dye) or maleimide-NOTA (a macrocyclic chelating agent for radiolabeling with ^18^F). The resulting conjugates, called **5a**-IRDye and **5a**-NOTA, could bind human and murine αvβ6 or αvβ8 with an affinity similar to that of **5a** (0.6-3 nM), suggesting that the peptide retains its integrin-binding properties after conjugation. Peptide **5a** could also recognize αvβ6 expressed on cell membranes, as suggested by the observation that **5a**-IRDye could recognize αvβ6-positive cells (but not αvβ6-negative cells) *in vitro*, in a manner that was inhibited by a neutralizing anti-αvβ6 antibody. The binding to cells was also competed by an excess of **5a** or A20FMDV2 (a known ligand of αvβ6), but not by the control peptide **2a** (containing RGE instead of RGD), suggesting that **5a** recognizes the RGD-binding site of αvβ6.

The uptake of **5a**-IRDye by BxPC-3 PDAC tumors *in vivo* was inhibited by an excess of **5a,** suggesting that also the tumor-homing properties of this compound depend on a receptor-mediated targeting mechanism. This hypothesis is further supported by the observation that a) tumor uptake of **5a**-IRDye was higher than that of **Cys**-IRDye or **5a**-**Scr**-IRDye, the latter consisting of a conjugate prepared with a scrambled sequence of **5a**, and b) tumor uptake of **5a**-NOTA-^18^F was significantly inhibited by an excess of **5a**.

*On*- and *off*-target peptide accumulation studies performed in the orthotopic model of αvβ6^+^/αvβ8^-^ pancreatic cancer showed good uptake of **5a**-IRDye in tumors but not in heart, brain, muscle, bone, spleen, intestine, and stomach. Some degree of dye uptake was also observed in liver, lung, and kidney. The uptake in the kidney might be due, in part, to a specific targeting mechanism, considering that mural mesangial cells can express αvβ8 11. However, the notion that αvβ6 expression in mouse kidney is negligible 27, and the observation that **5a**-IRDye and control **5a**-**Scr**-IRDye accumulated in this organ to similar extents suggests that most uptake in the kidney was related to renal clearance and not to a specific targeting mechanism. The uptake of **5a**-IRDye by the lung was possibly related to the fact that this organ expresses αvβ6 and αvβ8 integrins 28, 29.

Biodistribution studies performed with **5a**-NOTA-^18^F showed a pattern similar to that of **5a**-IRDye, except for some additional uptake in the stomach and femur. Of note, uptake of** 5a**-NOTA-^18^F in the lung and kidney, but not in the femur, was significantly inhibited by an excess of **5a**. The fact that the uptake in the femur was not blocked by the free peptide is probably due to the presence of some Al^18^F in our preparation or to a partial release of ^18^F from **5a**-NOTA-^18^F, known to accumulate in the bone 30. Interestingly, other investigators have shown that similar radiopeptides specific for αvβ6 accumulate in the kidney, stomach, and intestine 27, 31. In these studies, uptake in the stomach and intestine was attributed to the expression of αvβ6 in these organs 19, 27, whereas the uptake in kidney was related to an αvβ6-independent mechanism 27, 32, as also suggested by the observation that the accumulation in this organ was significantly blocked by administration of a scrambled sequence 27. In summary, the *on*- and *off*-target accumulation studies of **5a** in PDAC-bearing mice reveal specific accumulation in αvβ6-positive tumors and *off-*target accumulation in lung, liver, intestine, and kidney, with the last three organs likely mostly related to the excretory pathways of the peptide or its metabolites. On the other hand, the results of *in vitro* studies showing that **5a** can specifically bind αvβ6^-^/αvβ8^+^ cells (in an αvβ8-dependent manner) and the results of *in vivo* studies showing specific accumulation of **5a**-IRDye in αvβ6^-^/αvβ8^+^ prostate tumors (TRAMP-C2 model) suggest that this peptide can also accumulate on cells expressing this integrin in tumors, such as carcinoma cells and infiltrating regulatory T cells 12, 23, 33.

These results pave the way for several potential applications of peptide **5a,** including: a) diagnostic molecular imaging of αvβ6^+^ tumors with **5a**-NOTA-^18^F, b) fluorescence-guided surgery of tumors with **5a**-IRDye, and c) the delivery of other diagnostic, theranostic or therapeutic agents to tumors (such as antibodies, cytokines, drugs, nanoparticles, DNA complexes, and other compounds). Considering that different tumor types may express both αvβ6 and αvβ8 integrins (e.g., oral squamous cell carcinoma) the dual-receptor-targeting properties of **5a** provide an advantage over other mono-targeting compounds developed to date, as this might improve the tumor-targeting sensitivity and/or detection efficiency. This possibility should be further investigated in appropriate models. An additional advantage could be related to the fact that **5a**, being derived from a human protein (chromogranin A), could be less immunogenic than other peptides carrying viral sequences or containing non-natural amino acids, previously described.

Finally, the fact that **5a** can recognize the LAP-TGFβ complex-binding site of both αvβ6 and αvβ8 integrins opens the way to another potential application of this peptide in cancer therapy, i.e., targeting TGFβ activation in the tumor microenvironment. Indeed, previous NMR and computational/biochemical studies showed that the stapled CgA-derived peptide binds the RGD binding site of αvβ6 with receptor-ligand interactions similar to those observed for the proTGFβ1/αvβ6 complex 25, thereby suggesting that **5a** can block the binding of the inactive LAP-TGFβ complex to this integrin and, consequently, its activation. This view is supported the results of the present study, showing that **5a** can block the binding of αvβ6 and αvβ8 to isoDGR-peroxidase, a probe for the RGD-binding site of integrins 25, and inhibit TGFβ activation by αvβ8^+^ TRAMP-C2 cells. Thus, this peptide may represent a sort of bi-selective inhibitor of both αvβ6 and αvβ8 endowed of tumor-homing properties. Interestingly, recent studies have shown that regulatory T cells colonizing tumors express higher levels of αvβ8 than those isolated from lymphoid organs, and that these cells, by activating TGFβ in tumors, induce immunosuppression and favor tumor growth 23, 33. Other studies have shown that even αvβ8^+^ tumor cells can activate TGFβ and favor tumor growth in murine models 12. Notably, anti-αvβ8 antibodies capable of blocking TGFβ activation can induce tumor regression 12, 23. Similarly, anti-αvβ6 antibodies can inhibit the growth of αvβ6-positive tumors in mice through a TGFβ-regulated mechanism 24. Thus, considering that both αvβ6 and αvβ8 can activate TGFβ, both integrins may represent promising targets for cancer immunotherapy. Based on these concepts, a major novelty of the present study lies in the fact that **5a** might be considered as an efficient bispecific "inhibitor" of both αvβ6 and αvβ8 with the peculiarity of being also a tumor-homing compound. However, this hypothesis needs to be demonstrated in appropriate animal models. Furthermore, considering the short plasma half-life of **5a**-IRDye (~8 min) **(Figure S13)**, the development of derivatives with longer half-life, e.g., PEGylated-**5a**, is likely necessary to enable sustained blockade of αvβ6/αvβ8 in tumors.

Of note, another dual αvβ6/αvβ8 peptide ligand has been recently described (RGD-Chg-(*N*Me)E]-CONH_2_) 34. However, the tumor homing properties of this compound - which inhibits the binding of soluble integrin αvβ6 and αvβ8 to an immobilized ECM protein with an IC_50_ of 1.6 and 60 nM, respectively - have not been demonstrated.

## Conclusion

The results demonstrate that **5a** can home to αvβ6- and αvβ8-positive tumors. This compound can be exploited as a tumor-homing ligand for delivering imaging and anticancer compounds to αvβ6/αvβ8 single- or double-positive tumors. Furthermore, considering that **5a** can block the active site of αvβ6/αvβ8 and inhibit TGFβ activation (a potent immunosuppressive mechanism in tumors), our results suggest that this peptide is a peculiar tumor-homing bi-selective inhibitor of αvβ6 and αvβ8 that could be exploited for targeting this immunosuppressive mechanism in tumors.

## Supplementary Material

Supplementary materials and methods, figures and tables.

## Figures and Tables

**Table 1:** Binding affinity of chromogranin A-derived peptides and A20FMDV2 for human and murine αvβ6 and αvβ8 integrin.

Compound	Code		*Ki* ^b^ (competitive integrin binding assay)					
*Free peptide* ^ a^			*n* ^c^	Human αvβ6 (nM)			*n*	Human αvβ8 (nM)
CFETLRGEERILSILRHQNLLKELQD*-CONH_2_*	2a		2	>50000 ^d^			1	>50000 ^d^
*ac-*FETLRGDLRILSILRX_1_QNLX_2_KELQD*-CONH_2_*	5		7	0.6 ± 0.1 ^d^			6	3.2 ± 1.2^ d^
CFETLRGDLRILSILRX_1_QNLX_2_KELQD*-CONH_2_*	5a		5	1.70 ± 0.26				*NA^e^*
CIRLDLELINFQSDLQHELLKTRLRG	5a-Scr		1	>>200				*NA*
NAVPNLRGDLQVLAQKVART	A20FMDV2		8	0.9 ± 0.2^ d^			6	69 ± 0.2^ d^
					
*Peptide-NOTA conjugate*								
2a-NOTA			1	>>1000				*NA*
5a-NOTA			1	2.3				*NA*
	*EC_50_*^f^ (direct integrin binding assay)							
	αvβ6				αvβ8			
*Peptide-IRDye conjugate*	*n*	Human (nM)	*n*	Murine (nM)	*n*	Human (nM)	*n*	Murine (nM)
2a-IRDye	2	>>100	3	>>100	2	>>100	3	>>100
5a-IRDye	2	2.8 ± 0.05	3	0.9 ± 0.35	2	1.9 ± 0.37	3	0.65 ± 0.50
Cys-IRDye	2	>>100	3	>>100	2	>>100	3	>>100
5a-Scr-IRDye		*NA*		*NA*		*NA*		*NA*

**(a)** Single letter code; triazole-stapled residues (**X_1_** and **X_2_**, propargylglycine and azidolysine, respectively); *ac*-, N-terminal acetylated; *-CONH_2_,* C-terminal amidated. **(b)** *Ki*, inhibitory constant. Mean ± SE. **(c)** *n*, number of independent experiments. **(d)** Reprinted with the permission of Ref. 25. **(e)** *NA*, not analyzed. **(f)** *EC_50_*, Effective Concentration 50. Mean ± SE.

## Abbreviations

CgA: chromogranin A
TGFβ: transforming growth factor-β
LAP: latency associated peptide
MS: mass spectrometry
PET: positron emission tomography
CT: computed tomography
