# Supplementary material for: A stapled chromogranin A-derived peptide homes in on tumors that express αvβ6 or αvβ8 integrins
Source: Int J Biol Sci. 2023 Jan 1;19(1):156–66. doi: 10.7150/ijbs.76148 (PMC9760430; doi:10.7150/ijbs.76148)
Supplement: Supplementary file 1 — Supplementary materials and methods, figures and tables. [file ijbsv19p0156s1.pdf]

Supporting Information

A stapled chromogranin A-derived peptide homes in on tumors that express αvβ6 or αvβ8 integrins

Matteo Monieri, Paolo Rainone, Angelina Sacchi, Alessandro Gori, Anna Maria Gasparri, Angela Coliva, Antonio Citro, Benedetta Ferrara, Martina Policardi, Silvia Valtorta, Arianna Pocaterra, Massimo Alfano, Dean Sheppard, Lorenzo Piemonti, Rosa Maria Moresco, Angelo Corti*, and Flavio Curnis*

Supplemental Materials and Methods

Reagents

Recombinant human and murine αvβ6 and αvβ8 (Bio-techne); Human TGFβ (InvivoGen, cat. rcyc-htgfb1); IRDye® 800CW maleimide (LI-COR, cat. 929-80020); 1,4,7-triazacyclononane-1,4-bis-acetic acid-7-maleimidoethylacetamide (maleimide-NOTA, CAS number: 1295584-83-6, CheMatech, cat. C101; purity >95%); mouse anti-human/mouse αvβ6 antibody, clone 10D5, IgG2a (Millipore, cat. MAB2077Z); rabbit anti-human αvβ8 monoclonal antibody, clone EM13309, IgG (Absolute Antibodies, cat. Ab00892); mouse anti-human/mouse αvβ8 antibody, clone ADWA-11 [1]; control isotype-matched murine IgG_1, clone MOPC-31C (Sigma, cat. M9035); mouse anti-human CgA, clone 5A8 [2]; control isotype rabbit IgG (Abcam, cat. ab37415); Alexa Fluor 488-labeled goat anti-mouse (Invitrogen, cat. A-11001) and goat anti-rabbit (Invitrogen, cat. A-11034).

Cell lines

Human BxPC-3 pancreatic ductal adenocarcinoma (cat. CRL-168), human 5637 bladder carcinoma (cat. HTB-9), human MeWo melanoma (cat. HTB-65) and murine TRAMP-C2 prostate cancer (cat. CRL-2731) cells were from ATCC (American Type Culture Collection). Human BHY oral squamous carcinoma cells were from DSMZ (German Collection of Microorganisms and Cell Cultures, cat. ACC 404). Murine 5M7101 pancreatic cancer cells,

isolated from spontaneous liver metastases of a KCPhet mouse (Kras mutant; Pdx-1-Cre; Trp53 heterozygous null) were a kind gift from Dr. Hana Algül (Technische Universität München, Germany). BxPC-3 and 5637 were cultured in RPMI-1640 medium with standard supplements. 5M7101, BHY and TRAMP-C2 cells were cultured in DMEM medium containing standard supplements with an additional 1% non-essential aminoacids added for 5M7101 cells. MeWo cells were cultured in MEM medium with standard supplements with the addition of 1 % non-essential aminoacids and sodium pyruvate.

Human umbilical vein endothelial cells (HUVECs) were purchased from Lonza (cat. C2519A) and cultured as recommended by the manufacturer. One vial from a working cell bank was used to start with new experiments and cells were cultured for no longer than 4 weeks before use. All cell lines were free of mycoplasma as routinely tested with the MycoAlert Control Set (Lonza).

Peptide synthesis, purification, and characterization

All peptides used in this study were prepared by chemical synthesis (*see* **Table 1**). Peptide **5a,** which corresponds to peptide **5** with an additional cysteine residue at its N-terminus (*see* **Figure S1** and **Table 1** for its amino acid sequence and chemical modification), was synthetized with an S-tBu protected cysteine by stepwise microwave-assisted Fmoc solid-phase peptide synthesis, cyclized by Cu^{I}-mediated catalysis and purified by reversed phase high performance liquid chromatography (RP-HPLC), as described previously [3]. The pH of the triazole-stapled peptide was adjusted to 7.4 using 1 M sodium hydroxide. The S-tBu protecting group was removed by adding to the product tris(2-carboxyethyl)phosphine (TCEP) in 0.4 M sodium phosphate buffer, pH 7.0 (final concentration, 0.1 M)). The reaction was monitored by RP-HPLC. The resulting final product with a free N-terminal cysteine was then purified by RP-HPLC using a Shimadzu Prominence HPLC system, equipped with a Shimadzu Prominence preparative UV detector, connected to Shimadzu Shim-pack G15 10 µm C18 90Å (250 x 20 mm). The column was eluted with mobile phase A (3% acetonitrile, 0.07% TFA in water) and mobile phase B (70% acetonitrile, 0.07% TFA in water) using the following chromatographic method: 0% B (7 min), 0–30% B linear-gradient (40 min); flow rate, 14 ml/min. Peptide purity was ≥ 95%, as determined by analytical RP-HPLC using a Shimadzu Shim-pack GWS 5 µm C18 90Å column (150 x 4.6 mm) connected to a diode array detector.

A peptide with RGE in place of RGD (called **2a**) and a scrambled sequence of peptide **5a** (without the triazole bridge, called **5a-Scr**, *see* **Table 1**) were also synthesized and purified as described above and used as controls.

Peptide A20FMDV2 (**Table 1**) was purchased from Biomatik (Delaware, USA).

The identity of each peptide was confirmed by electrospray ionization mass spectrometry analysis using an LTQ-XL Orbitrap spectrometer (Thermo Scientific) (*see* **Table S1**). The concentration of thiol-containing peptides was determined by the Ellman's assay, whereas the concentration of the other peptides was determined by infrared spectroscopy using a Direct Detect™ spectrophotometer (EMD, Millipore).

Cell adhesion assay

Cell adhesion assays were carried out using 96-well polyvinyl chloride microtiter plates (Carlo Erba, cat. FA5280100) as described previously [2].

TGFβ bioassay

The inhibitory activity of peptides on TGFβ activation by TRAMPC-2 cells was tested using HEK-Blue™ TGFβ cells (InvivoGen, cat. hkb-tgfbv2), a cell model that allows to detect bioactive TGFβ in TRAMP-C2 cell supernatant, by monitoring the activation of the TGFβ/Smad pathway with a Smad-inducible secreted embryonic alkaline phosphatase (SEAP) reporter. TRAMPC-2 cells were seeded in a 96-well cell culture plate (50,000/well, 50 µl in DMEM medium containing 10% FCS, and standard supplements (*cell culture medium*) and cultured for 2 h to allow cell attachment. Then 50 µl aliquots of peptide **5a** (or **2a)** solutions in *cell culture medium* (0.1 nM to 60 µM) was added and further incubated for 20 h. Aliquots of conditioned cell supernatants (20 µl) were then mixed with HEK-Blue™ TGFβ cell suspensions (50,000/well, 96-well plate, 180 µl) and incubated for 20 h. Human TGFβ solutions (range 6-5000 pg/ml) were used as reference standards. SEAP activity in the supernatant of HEK-Blue™ TGF-β cells was quantified using the SEAP substrate QUANTI-Blue™, as recommended by the kit manufacturer.

Conjugation of peptides to IRDye® 800CW fluorophore

Peptide **5a**, **2a** and **5a-Scr** were coupled to IRDye® 800 CW maleimide (IRDye) as follows: an aliquot of 1 mg peptide was dissolved in 0.5 ml of degassed 5 mM sodium phosphate buffer, pH 7.4, and mixed with 0.5 ml of a solution of IRDye (0.9 eq) in 50% aqueous acetonitrile (25% final acetonitrile concentration). The mixtures were incubated at room temperature. The coupling reactions were monitored using RP-HPLC and stopped upon completion. The mixtures were then purified by semipreparative RP-HPLC. The products were lyophilized, resuspended in 50% acetonitrile, and then diluted in water (7% acetonitrile, final concentration).

A control conjugate with a cysteine in place of the peptide was also prepared in parallel. The identity of the purified products, called **5a**-IRDye, **2a**-IRDye, and **Cys**-IRDye, was confirmed by mass spectrometry analysis (**Table S1**). In addition, the purity and identity of **5a**-IRDye was monitored by liquid chromatography–mass spectrometry (LC-MS). The results confirm that the product consist of a single peak at 230 and 735 nm with the expected molecular weight (purity >95%) (**Figure S3A**).

The simplified molecular-input line-entry system (SMILES) of **5a-**, **2a-**, **Cys**- and **5a-Scr**-IRDye are reported in **Table S2**.

The concentration of each conjugate was quantified by spectrophotometric analysis at 774 nm (molar absorption coefficient, 240,000 M^{-1}, cm^{-1}) (**Figure S3B**). The final yield of **5a**-IRDye was 300-400 μg.

Binding of peptide-IRDye conjugates to αvβ6 and αvβ8 integrin

The binding affinity of peptide-IRDye conjugates for human and murine αvβ6 and αvβ8 integrins was determined as follows: 96-well clear-bottom black microtiter plates (Corning® cat. 3601) were coated with 2 μg/ml αvβ6 or αvβ8 (50 μl/well), in Dulbecco's phosphate-buffered saline containing Ca^{2+} and Mg^{2+} (DPBS) and left to incubate overnight at 4°C. After washing, plates were incubated with 3% bovine serum albumin (BSA) in DPBS (200 μl/well, 1 h, at room temperature), washed again with 0.9% sodium chloride, and filled with various amounts of peptide-IRDye conjugates (range 0.01–100 nM) in 25 mM Tris-HCl, pH 7.4, containing 150 mM sodium chloride, 1 mM magnesium chloride, 1 mM manganese chloride, 0.05% Tween-20 and 1% BSA (100 μl/well). After 1 h of incubation, the plates were washed three times with the same buffer, without BSA. Bound fluorescence was then quantified by scanning the empty plates with an Odyssey CLx near-infrared fluorescence imaging system (LI-COR) using the following settings: scan intensity, *3*; scan focus offset*, 3*; scan quality, *highest*; channel, *800*; resolution*, 169 µm*.

Binding of peptide-IRDye conjugates to cultured living cells

The binding of peptide-IRDye conjugates to BxPC-3, 5M7101, and HUVECs cells was analyzed as follows. Cells were grown in black 96-well microtiterplates with clear-bottom (Corning® cat. 3603, 2–3×10^4 cells/well, seeded 48 h before the experiment). After washing twice with 0.9% sodium chloride solution, the cells were incubated with 25 mM Hepes buffer, pH 7.4, containing 150 mM sodium chloride, 1 mM magnesium chloride, 1 mM manganese

chloride and 1% BSA (*binding buffer*) for 5 min. Peptide-IRDye conjugates (0.32–200 nM in *binding buffer*) were then added to the cells and left to incubate for 1 h at 37 °C, 5% CO_2. After three washings with *binding buffer* (5 min each, 200 µl/well), the cells were fixed with PBS containing 2% paraformaldehyde and 3% sucrose for 15 min at room temperature. Binding of conjugates to cells was quantified by scanning the plate (filled with PBS, 100 µl/well) with the Odyssey CLx (LI-COR) using the above settings, except that the *scan focus offset* and *scan intensity* were set to 4 and 8.5, respectively. In some experiments, to quantify the cell number in each well, plates were incubated with 5 µg/ml of 4',6-diamidino-2-phenylindole (DAPI) for 15 min, washed twice with PBS and then read with a VICTOR3 plate reader (Perkin Elmer, Waltham, MA, USA), using the following filters: excitation, 355 ± 40 nm; emission, 460 ± 25 nm (acquisition, 1 s).

Flow cytometry analysis

Flow cytometry analysis of αvβ6 and αvβ8 cell-surface integrin expression was carried out as described previously [3].

Peptide conjugation to maleimide-NOTA

Peptides **5a** and **2a** were coupled to maleimide-NOTA as follows: 6.5 µmol of maleimide-NOTA in 0.445 ml of 10 mM phosphate buffer, pH 7.4, containing 138 mM sodium chloride, 2.7 mM potassium chloride (called PBS-SIGMA) was mixed with 3.21 µmol of peptide in 1.555 ml of PBS-SIGMA (maleimide-NOTA/peptide molar ratio, 2:1), left to react for 16 h at 4°C and mixed with 50% (vol/vol) orthophosphoric acid (100 µl). The conjugates were then purified using a semi-preparative RP-HPLC C18 column (LUNA, 250 x 10 mm, 100 angstrom, 10 µm, Phenomenex) connected to an AKTA Purifier 10 HPLC (GE Healthcare), as follows: mobile phase A, 0.1% trifluoroacetic acid (TFA) in water; mobile phase B, 0.1% TFA in 95% acetonitrile; 0% B (9 min), linear-gradient 0–100% B (40 min), 100% B (10 min), 0% B (10 min); flow rate, 5 ml/min. Fractions containing the conjugates were pooled, lyophilized, resuspended in water, and analyzed by analytical RP-HPLC using a C18 column (LUNA, 250 x 4.6 mm, 100 angstrom, 5 µm, Phenomenex), using the same method as described above except that the flow rate was 0.5 ml/min. The identity of the product was determined by mass spectrometry analysis (LTQ-XL Orbitrap, **Table S1** and **Scheme S2**). The SMILES of **5a**-NOTA and **2a**-NOTA are reported in **Table S2**. The final yields were ~ 8 mg.

^{18}F radiolabeling of 5a-NOTA conjugate

The peptide **5a**-NOTA conjugate was radiolabeled in-house with ^{18}F using a modified Tracerlab FX-N automatic module (GE Healthcare, Illinois, USA). Two-four GBq ^{18}F-sodium fluoride, produced using an IBA 18/9 MeV Cyclotron (IBA RadioPharma Solutions, Louvain-la-Neuve, Belgium), was delivered to the module and loaded onto an anion-exchanger cartridge (Sep-Pak Accell Plus QMA Plus Light, Waters, Italy) pre-conditioned with 0.5 M sodium acetate, pH 8.5 (10 ml), and washed with 10 ml of metal-free water. The column was washed with water (10 ml) and 0.9% sodium chloride (200 µl) and eluted with 0.9% sodium chloride (300 µl). Fluorine was collected into a glassy carbon reactor and mixed with metal-free 0.5 M sodium acetate, pH 4.2 (15 µl), 8.6 mg/ml **5a**-NOTA conjugate in water (15 µl), 2 mM aluminum chloride in 0.5 M sodium acetate, pH 4.2 (3.6 µl), 50 mM ascorbic acid in 0.5 M sodium acetate, pH 4.2 (5 µl) and pure ethanol (330 µl). The product was then incubated at 107°C for 15 min. After cooling, the product was diluted to 10 ml with deionized water, loaded onto a C18 cartridge (Sep-Pak Plus Waters), washed with water (12 ml), and eluted with ethanol/water (1:1) (1 ml). The product was then diluted to 5 ml with 0.9% sodium chloride. Radiochemical purity was checked by RP-HPLC using a C18 column (ACE C18, 250 x 4.6 mm, Phenomenex) connected to an HPLC system (Waters Corporation, Milford Massachusetts, USA) equipped with a radiochemical counter, under the following chromatographic conditions: buffer A, 0.1% TFA in water; buffer B, 0.1% TFA in acetonitrile; flow rate, 1 ml/min; linear-gradient 0-20 min: 20% B; 20-40 min: 85% B; 40-45 min: 85% B; 45-55 min: 0% B.

In vivo **studies in animal models**

Subcutaneous mouse tumor models of pancreatic and prostate cancer

Eight-week-old female NOD scid gamma (NSG) and nu/nu CD1 mice (Charles River Laboratories Italia S.p.A., Calco, Italy) were injected with $1x10^7$ BxPC-3 cells, resuspended in 100 ml of 0.9% sodium chloride, into the shoulder. Eight-week-old female C57BL/6N mice (Charles River Laboratories) were injected with 1.5×10^6 and 0.5×10^6 5M7101 cells in 100 µl 0.9% sodium chloride, into the left and right lower flank, respectively.

Nu/nu CD1 female and BALB/C RAG2$\gamma c^{-/-}$ male mice were injected with $5x10^7$ TRAMP-C2 cells, resuspended in 100 ml of 0.9% sodium chloride, into the shoulder. When the tumors reached a diameter of 0.3-0.6 cm, the mice were anesthetized with 2% isoflurane and subjected to imaging studies.

Orthotopic mouse model of pancreatic cancer.

BxPC-3 cells were orthotopically implanted into nu/nu female CD1 mice. For this purpose, $1x10^6$ cells in 60 µl of phosphate-buffered saline containing 25% growth factor-reduced Matrigel (Corning) were injected into the pancreas of mice as previously described [4]. Eighteen days later, the mice were anesthetized and underwent optical imaging studies.

Near-infrared imaging studies

Mice were injected with the peptide-IRDye conjugate (1-1.2 nmol in 100 µl of 50 mM sodium phosphate buffer, 150 mM sodium chloride, pH 7.4) into the tail vein. For blocking experiments, mice were injected intravenously with unlabeled peptide **5a** (130 nmol/mouse, in PBS containing 100 µg/ml human serum albumin) 10 min before **5a**-IRDye administration. Mice were imaged at various time point (range 0-72 h) using the IVIS Spectrum CT Imaging System (PerkinElmer) equipped with 745 nm excitation and 800 nm emission filters and the following instrument settings: exposure, auto; binning, 8; F/stop, 2; and field of view, C or D. Images were acquired with three-four mice simultaneously. In some experiments, the mice were sacrificed after the last scan, and the tumors and various organs were removed for *ex vivo* imaging. For the quantification of the fluorescence uptake by the exposed pancreas the left kidney was surgically removed before imaging. Regions of interest (ROI) were drawn on the images, and the average (Avg) radiant efficiency was calculated using the Living Image 4.3.1 software (PerkinElmer). To determine the uptake of fluorescence by tumors *in vivo*, we subtracted the signal from ROIs drawn on forepaws or hind paws, whereas for *ex vivo* biodistribution studies, we subtracted the autofluorescence of the tray on which the organs were located. Tumor-to-background ratio (TBR) was calculated using the normalized regions according to the following formula: normalized Avg Radiant Efficiency tumor/normalized Avg Radiant Efficiency background.

PET imaging and biodistribution studies

For kinetics studies, the **5a**-NOTA-^{18}F conjugate was injected into the tail vein of BxPC-3 tumor-bearing mice 30-35 days after tumor cell implantation (~ 4 MBq/mice, in 100 µl of water containing <10% ethanol). The radiotracer uptake was monitored by whole-body positron emission tomography (PET)/computed tomography (CT) using the preclinical β-cube® and X-cube® scanners (Molecubes, Gent, BE), respectively. Three mice were placed side-by-side in a prone position under anesthesia (2% isoflurane in medical air) and imaged after 1, 2, and 4 h. For the blocking study, tumor-bearing mice (n=3) were intravenously injected with unlabeled peptide **5a** (130 nmol/mouse, in PBS containing 100 µg/ml human serum albumin), 10 min

before the administration of **5a**-NOTA-^{18}F (~ 3 MBq/mice**)**. After 2 h, mice were whole-body PET/CT imaged and sacrificed for *ex-vivo* quantification of radiotracer uptake. For *ex vivo* biodistribution, the mice were euthanized by cervical dislocation. Tumor and selected organs were collected, rinsed, weighted, and analyzed for their radioactivity content using a γ-counter (LKB Compugamma CS 1282). CT and PET images were reconstructed using the proprietary Molecubes software included in the system. CT images were reconstructed with a 200 μm isotropic pixel size using a standard ISRA algorithm. PET images were reconstructed using a List-Mode OSEM algorithm with 30 iterations and 400 μm isotropic voxel size, accounting for the tracer decay correction. CT/PET images were processed by Region of Interest (ROI) analysis using PMOD software v.4.1 (Zurich, Switzerland). The uptake of radioactivity is expressed as "standardized uptake value" (SUV) according to the formula: tumor concentration activity [MBq/g] / (injected activity [MBq]/animal weight [g]). Maximum (SUV max) and mean (SUV mean) uptake values were calculated in kinetics and blocking studies, respectively. The radioactivity concentration in biodistribution study is reported as percentage of injected dose per gram of tissue (%ID/g). Results and images are also showed as tumor-to-muscle ratio (T/M).

Table S1: Molecular mass of synthetic peptides and conjugates, as determined by Electrospray Ionization (ESI) mass spectrometry (MS) analysis.

Compound	*Monoisotopic mass (Da)*	
	Expected	Found
Peptide (code)		
**2a**	3151.69	3151.69
**5**	3059.68	3059.75
**5a**	3120.69	3120.70
**5a-Scr**	3122.70	3122.73
**A20FMDV2**	2162.23	2162.30
Peptide-IRDye conjugate		
**2a-**IRDye	4275.98	4275.98
**5a**-IRDye	4244.99	4245.00
**Cys-**IRDye	1245.48	1245.30
Peptide-NOTA conjugate		
**2a**-NOTA	3576.10	3576.88
**5a**-NOTA	3545.88	3545.89

Table S2: SMILES codes of the peptide-IRDye and peptide-NOTA conjugates.

	SMILES CODE [a]
Peptide-IRDye conjugate	
**2a**-IRDye	CCC(C)C(NC(=O)C(CO)NC(=O)C(CC(C)C)NC(=O)C(NC(=O)C(CCCNC(=N)N)NC(=O)C(CCC(=O)O)NC(=O)C(CCC(=O)O)NC(=O)CNC(=O)C(CCCNC(=N)N)NC(=O)C(CC(C)C)NC(=O)C(NC(=O)C(CCC(=O)O)NC(=O)C(Cc1ccccc1)NC(=O)C(N)CSC8CC(=O)N(CCNC(=O)CCCCCN7/C(=C\C=C/4CCCC(/C=C/C3=[N+](CCCCS(=O)(=O)O)c2ccc(S(=O)(=O)[O])cc2C3(C)C)=C4Oc5ccc(S(=O)(=O)O)cc5)C(C)(C)c6cc(S(=O)(=O)O)ccc67)C8=O)C(C)O)C(C)CC)C(=O)NC(CC(C)C)C(=O)NC(CCCNC(=N)N)C(=O)NC(Cc9c[nH]cn9)C(=O)NC(CCC(N)=O)C(=O)NC(CC(N)=O)C(=O)NC(CC(C)C)C(=O)NC(CC(C)C)C(=O)NC(CCCCN)C(=O)NC(CCC(=O)O)C(=O)NC(CC(C)C)C(=O)NC(CCC(N)=O)C(=O)NC(CC(=O)O)C(N)=O
**5a**-IRDye	CCC(C)C(NC(=O)C(CO)NC(=O)C(CC(C)C)NC(=O)C(NC(=O)C(CCCNC(=N)N)NC(=O)C(CC(C)C)NC(=O)C(CC(=O)O)NC(=O)CNC(=O)C(CCCNC(=N)N)NC(=O)C(CC(C)C)NC(=O)C(NC(=O)C(CCC(=O)O)NC(=O)C(Cc1ccccc1)NC(=O)C(N)CSC8CC(=O)N(CCNC(=O)CCCCCN7/C(=C\C=C/4CCCC(/C=C/C3=[N+](CCCCS(=O)(=O)O)c2ccc(S(=O)(=O)[O-])cc2C3(C)C)=C4Oc5ccc(S(=O)(=O)O)cc5)C(C)(C)c6cc(S(=O)(=O)O)ccc67)C8=O)C(C)O)C(C)CC)C(=O)NC(CC(C)C)C(=O)NC(CCCNC(=N)N)C(=O)NC9Cc%10cn(CCCCC(C(=O)NC(CCCCN)C(=O)NC(CCC(=O)O)C(=O)NC(CC(C)C)C(=O)NC(CCC(N)=O)C(=O)NC(CC(=O)O)C(N)=O)NC(=O)C(CC(C)C)NC(=O)C(CC(N)=O)NC(=O)C(CCC(N)=O)NC9=O)nn%10
**Cys**-IRDye	CC7(C)/C(=C/C=C\3CCCC(/C=C/C2=[N+](CCCCS(=O)(=O)O)c1ccc(S(=O)(=O)[O-])cc1C2(C)C)=C3Oc4ccc(S(=O)(=O)O)cc4)N(CCCCCC(=O)NCCN5C(=O)CC(SC[C@H](N)C(=O)O)C5=O)c6ccc(S(=O)(=O)O)cc67
**5a-Scr**-IRDye	CC[C@H](C)[C@H](NC(=O)[C@@H](N)CSC6CC(=O)N(CCNC(=O)CCCCCN5/C(=C\C=C/2CCCC(/C=C/C1=[N+](CCCCS(=O)(=O)O)/C/C=C\C(S(=O)(=O)[O-])=C/CC1(C)C)=C2Oc3ccc(S(=O)(=O)O)cc3)C(C)(C)c4cc(S(=O)(=O)O)ccc45)C6=O)C(=O)N[C@@H](CCCNC(=N)N)C(=O)N[C@@H](CC(C)C)C(=O)N[C@@H](CC(=O)O)C(=O)N[C@@H](CC(C)C)C(=O)N[C@@H](CCC(=O)O)C(=O)N[C@@H](CC(C)C)C(=O)N[C@H](C(=O)N[C@@H](CC(N)=O)C(=O)N[C@@H](Cc7ccccc7)C(=O)N[C@@H](CCC(N)=O)C(=O)N[C@@H](CO)C(=O)N[C@@H](CC(=O)O)C(=O)N[C@@H](CC(C)C)C(=O)N[C@@H](CCC(N)=O)C(=O)N[C@@H](Cc8c[nH]cn8)C(=O)N[C@@H](CCC(=O)O)C(=O)N[C@@H](CC(C)C)C(=O)N[C@@H](CC(C)C)C(=O)N[C@@H](CCCCN)C(=O)N[C@H](C(=O)N[C@@H](CCCNC(=N)N)C(=O)N[C@@H](CC(C)C)C(=O)N[C@@H](CCCNC(=N)N)C(=O)NCC(=O)O)[C@@H](C)O)[C@@H](C)CC
Peptide-NOTA conjugate	
**2a**-NOTA	CC[C@H](C)[C@H](NC(=O)[C@H](CO)NC(=O)[C@H](CC(C)C)NC(=O)[C@@H](NC(=O)[C@H](CCCNC(=N)N)NC(=O)[C@H](CCC(=O)O)NC(=O)[C@H](CCC(=O)O)NC(=O)CNC(=O)[C@H](CCCNC(=N)N)NC(=O)[C@H](CC(C)C)NC(=O)[C@@H](NC(=O)[C@H](CCC(=O)O)NC(=O)[C@H](Cc1ccccc1)NC(=O)[C@@H](N)CSC3CC(=O)N(CCNC(=O)CN2CCN(CC(=O)O)CCN(CC(=O)O)CC2)C3=O)[C@@H](C)O)[C@@H](C)CC)C(=O)N[C@@H](CC(C)C)C(=O)N[C@@H](CCCNC(=N)N)C(=O)N[C@@H](Cc4c[nH]cn4)C(=O)N[C@@H](CCC(N)=O)C(=O)N[C@@H](CC(N)=O)C(=O)N[C@@H](CC(C)C)C(=O)N[C@@H](CC(C)C)C(=O)N[C@@H](CCCCN)C(=O)N[C@@H](CCC(=O)O)C(=O)N[C@@H](CC(C)C)C(=O)N[C@@H](CCC(N)=O)C(=O)N[C@@H](CC(=O)O)C(N)=O
**5a**-NOTA	CC[C@H](C)[C@H](NC(=O)[C@H](CO)NC(=O)[C@H](CC(C)C)NC(=O)[C@@H](NC(=O)[C@H](CCCNC(=N)N)NC(=O)[C@H](CC(C)C)NC(=O)[C@H](CC(=O)O)NC(=O)CNC(=O)[C@H](CCCNC(=N)N)NC(=O)[C@H](CC(C)C)NC(=O)[C@@H](NC(=O)[C@H](CCC(=O)O)NC(=O)[C@H](Cc1ccccc1)NC(=O)[C@@H](N)CSC3CC(=O)N(CCNC(=O)CN2CCN(CC(=O)O)CCN(CC(=O)O)CC2)C3=O)[C@@H](C)O)[C@@H](C)CC)C(=O)N[C@@H](CC(C)C)C(=O)N[C@@H](CCCNC(=N)N)C(=O)N[C@H]4Cc5cn(CCCC[C@@H](C(=O)N[C@@H](CCCCN)C(=O)N[C@@H](CCC(=O)O)C(=O)N[C@@H](CC(C)C)C(=O)N[C@@H](CCC(N)=O)C(=O)N[C@@H](CC(=O)O)C(N)=O)NC(=O)[C@H](CC(C)C)NC(=O)[C@H](CC(N)=O)NC(=O)[C@H](CCC(N)=O)NC4=O)nn5

a) The SMILES were generated using the following software: http://www.cheminfo.org/flavor/malaria/Utilities/SMILES_generator___checker/index.html

A

Peptide **5a**: CFETLRGDLRILSILR***X₁***QNL***X₂***KELQD$_{CONH_2}$

B

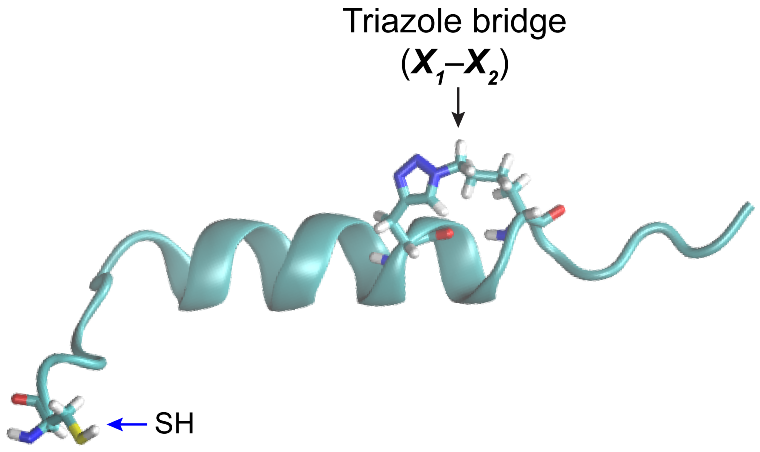

Figure S1. Aminoacidic sequence and 3D-model of peptide 5a.

A) Amino acid sequence of peptide **5a** derived from the 38-63 region of human chromogranin A. The one-letter amino acid code was used; $\mathbf{X_1}$ and $\mathbf{X_2}$ correspond to the propargylglycine and azidolysine residues, respectively, which form a triazole bridge after the click chemistry reaction; *CONH₂* represents the amidated C-terminus of the peptide.

B) Representative 3D model of peptide **5a**. The model was generated by modifying one of the NMR bundle structures of $CgA_{39\text{-}63}$ (PDB code: 6R2X) [3] by adding the triazole-bridged macrocyclic scaffold between residues in position $\mathbf{X_1}$ and $\mathbf{X_2}$. Furthermore, an additional N-terminal cysteine (*blue arrow*) was added and used for chemical coupling to maleimide-containing imaging compounds. Finally, the structure was minimized using Schrodinger Suite software (Schrodinger, LCC, New York, NY, 2019). *Black arrow* indicates the triazole bridge inserted to stabilize the alpha-helical structure of the peptide.

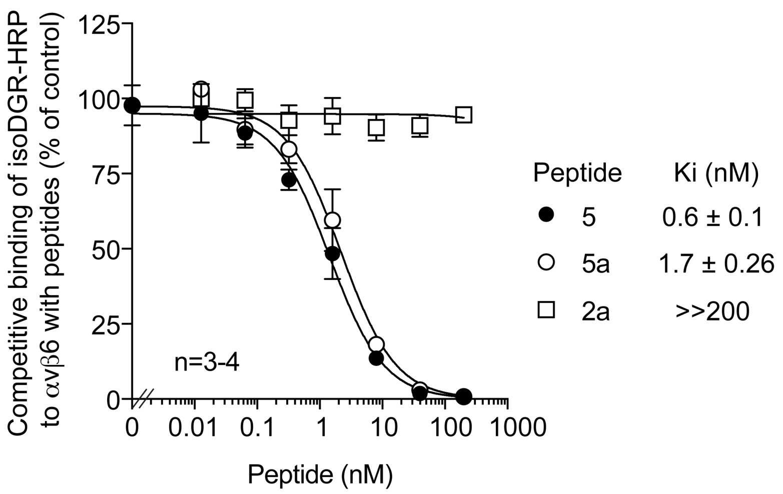

Figure S2. Competitive binding of *iso*DGR-peroxidase conjugate with peptide 5, 5a and 2a to αvβ6-coated microtiter plates.

The competitive binding assay was performed as previously described [3], using an isoDGR peptide (an RGD mimetic) labeled with peroxidase (*iso*DGR-HRP) as a probe for the integrin-binding site. A representative experiment (mean ± SE of 3-4 technical replicates) is shown. The resulting *Ki* values are shown (mean ± SE of n=3-7 independent experiments, *see also* **Table 1**).

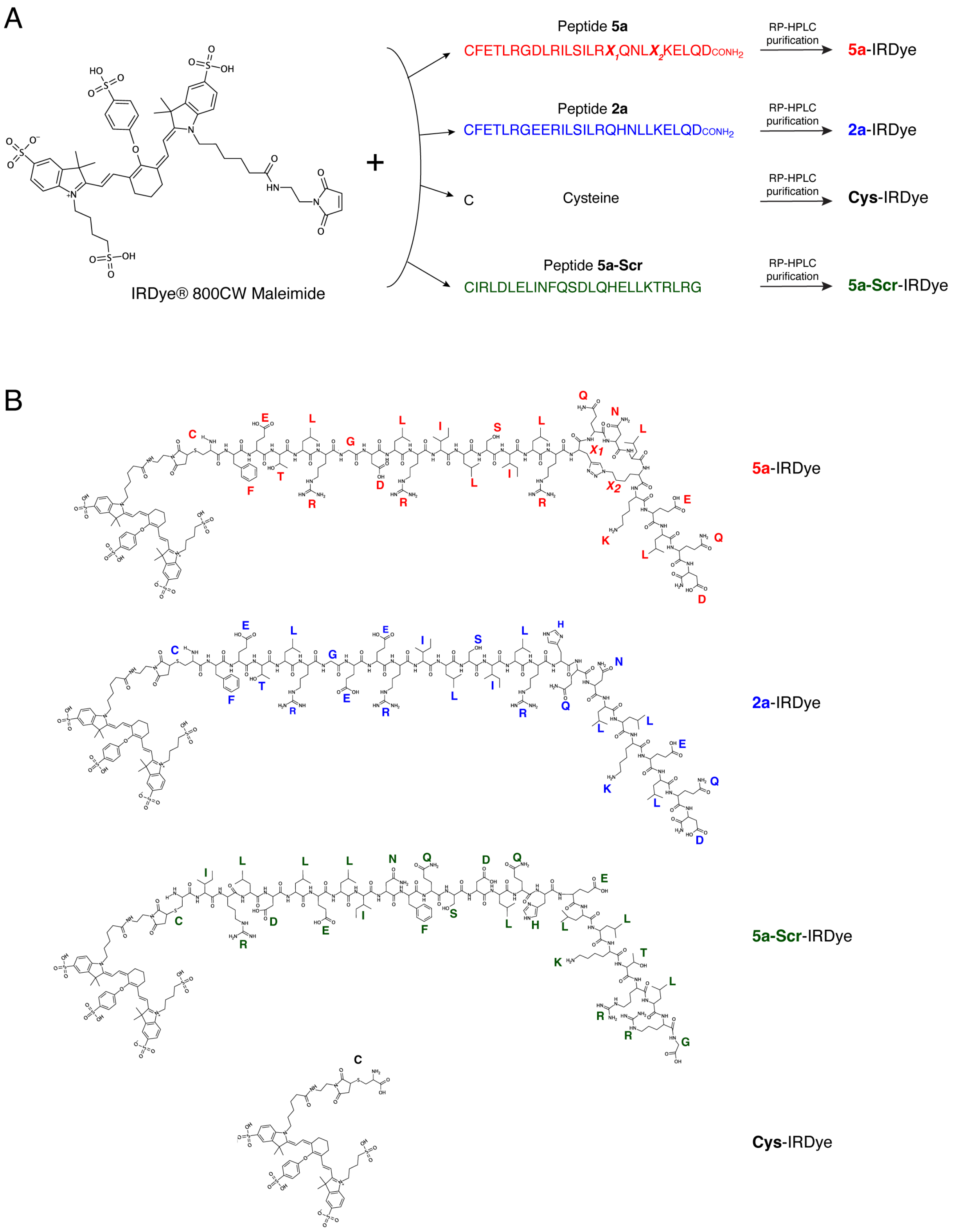

Scheme 1. Schematic representation of peptide-IRDye conjugate preparation (A) and conjugate structures (B).

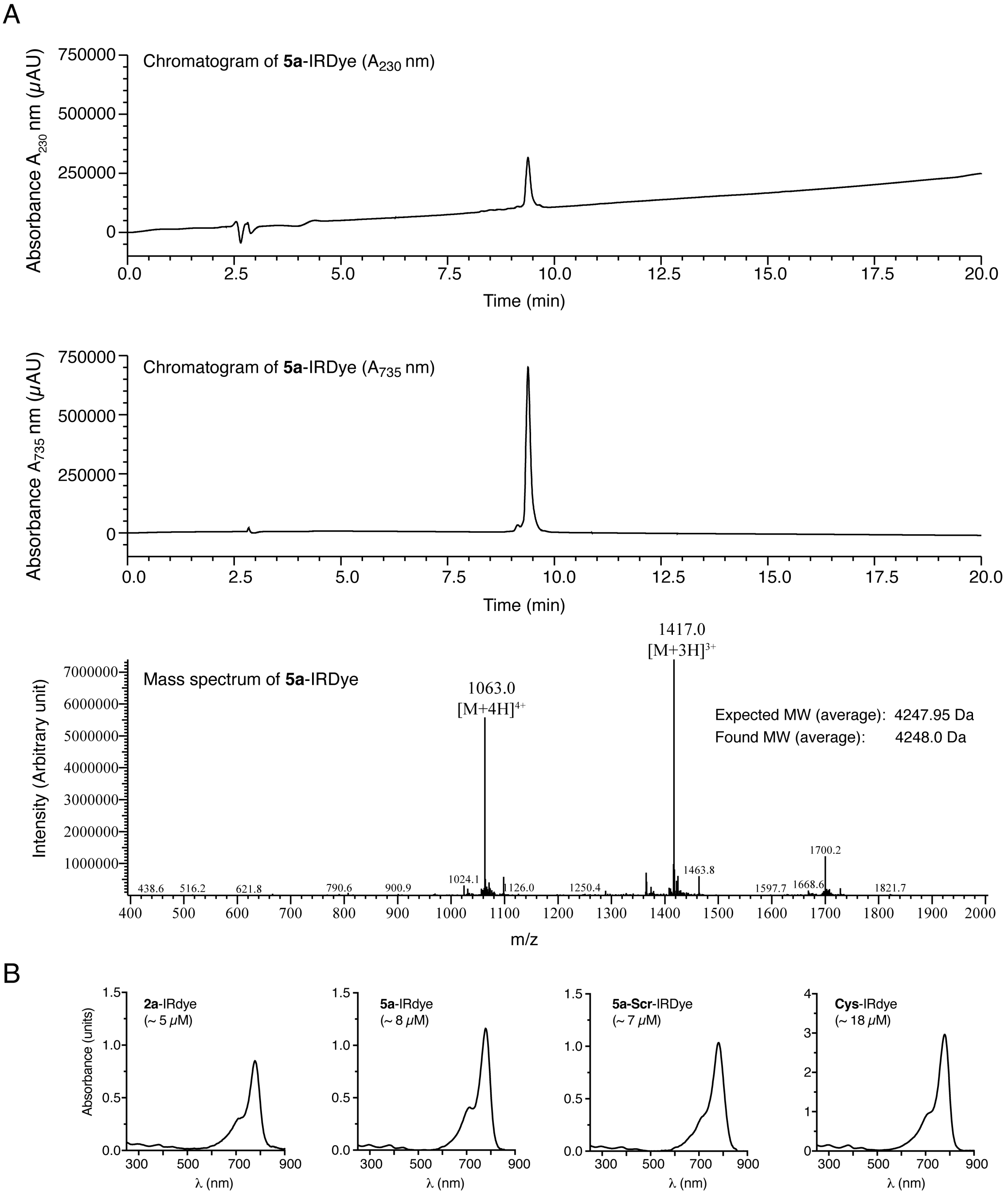

Figure S3. Characterization of peptide-IRDye conjugates by UV-IR spectrophotometric and liquid-chromatography/mass spectrometry (LC-MS) analysis. A) LC-MS of **5a**-IRDye. The **5a**-IRDye (~ 10 µg) was analyzed by LC-MS using a Kinetex C18 column (100Å, 100 x 4.6 mm, Phenomenex) connected to Shimadzu Prominence HPLC system equipped with a PDA detector and a LCMS-2020 module (Shimadzu), as follows: mobile phase A, 0.1% formic acid in water; mobile phase B, 0.1% formic acid in 100% acetonitrile; 0–100% B (linear-gradient 16 min), flow rate, 0.4 ml/min. MS acquisition range: 500-2000 Da (2000 Da, upper instrumental limit). **B**) Absorption spectra of the indicated peptide-IRDye conjugates in PBS.

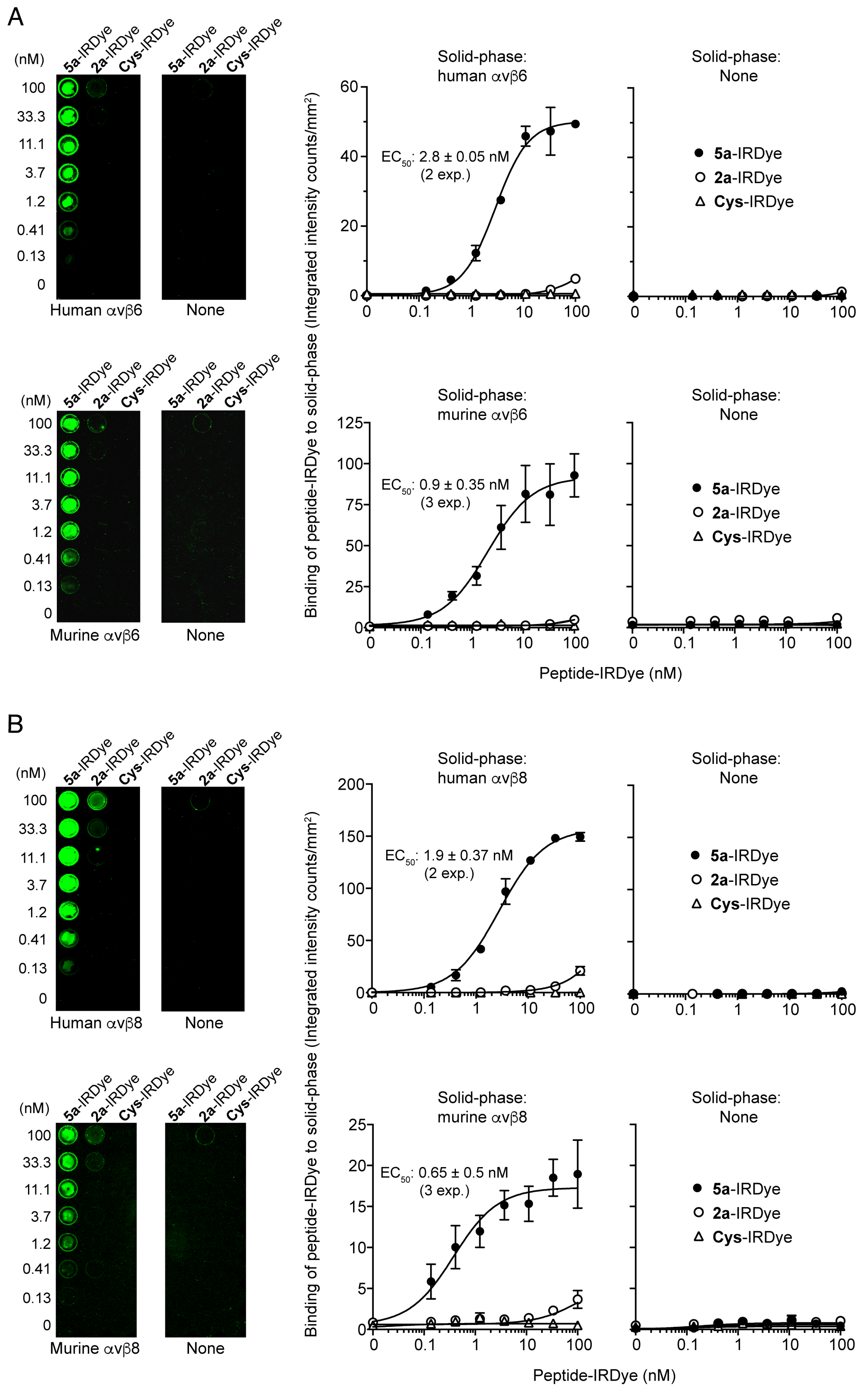

Figure S4. Binding of peptide-IRDye conjugates to human or murine αvβ6- and αvβ8 -coated microtiter plates.

Binding of **5a-**, **2a-** and **Cys-**IRDye to microtiterplates coated with or without recombinant human (4 µg/ml) or murine (2 µg/ml) αvβ6 (**A**) and αvβ8 (**B**). Various amounts of the

conjugates were added to the microtiterplates and incubated at room temperature for 1 h (see *Experimental Section*). After washing, bound fluorescence was measured using an Odyssey CLx (LI-COR) scanner. Representative images of the scanned plates and quantification of binding are shown. Mean ± SE of triplicate wells. The *Effective Concentration 50* (EC_{50}, mean ± SEM) of the indicated number of independent experiments (*exp.*) is shown.

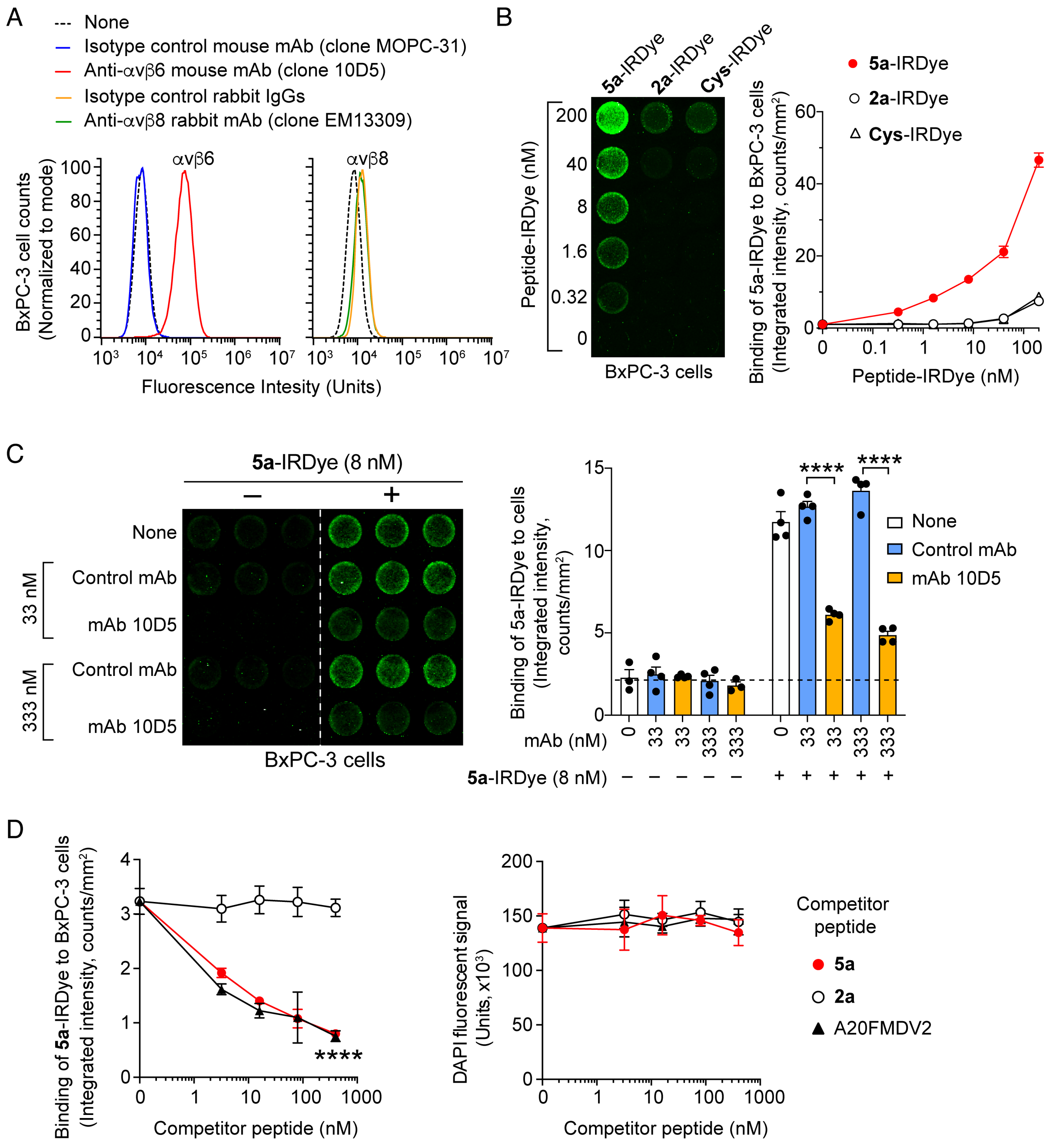

Figure S5. The binding of 5a-IRdye to BxPC-3 cells is mediated by peptide 5a and blocked by a neutralizing anti-αvβ6 mAb.

A) Expression of αvβ6 and αvβ8 by BxPC-3 cells assessed by FACS analysis using the indicated monoclonal antibodies, followed by an AlexaFluor 488-goat anti-mouse or anti-rabbit IgG polyclonal antibody.

B) Binding of the indicated peptide-IRDye conjugates to BxPC-3 cells. Various amounts of the conjugates were added to cell monolayers (grown in 96-well microtiter plates) and incubated at 37 °C, 5% CO_2 for 1 h. After washing, bound fluorescence was measured using an Odyssey

CLx (LI-COR) scanner. A representative image of the scanned plate and a graph reporting all binding data are shown. Mean ± SE of triplicate wells.

C) Competition of **5a**-IRDye binding to BxPC-3 cells with mAb 10D5 (a neutralizing anti-αvβ6 mAb) or a matched isotype control antibody (clone MOPC-31). The indicated amounts of mAbs were mixed with or without **5a**-IRDye (8 nM) and added to BxPC-3 cells prepared as described above. After 1 h, the plates were washed, and bound fluorescence was quantified as described above. A representative image of the scanned plate and a graph reporting binding data are shown. Mean ± SE of (n=3-4 wells). *Dotted line* shows the autofluorescence. ****, P<0.0001 by *two-tail t-test.*

D) Effect of unlabeled **5a**, **2a,** and A20FMDV2 peptides on **5a**-IRDye binding to BxPC-3 cells. Various amounts of unlabeled peptides were mixed with **5a**-IRDye (4 nM) and added to the cells. After 1 h, the plate was washed, and bound fluorescence was quantified as described above (*left panels*). Cells were then stained with DAPI (a nuclear stain) to quantify the total number of cells and fluorescence was measured using a fluorescence plate reader (*right panels*). Mean ± SE (n=3-4 wells). ****, P<0.0001 by *two-tail t-test* (**5a** *vs* **2a**).

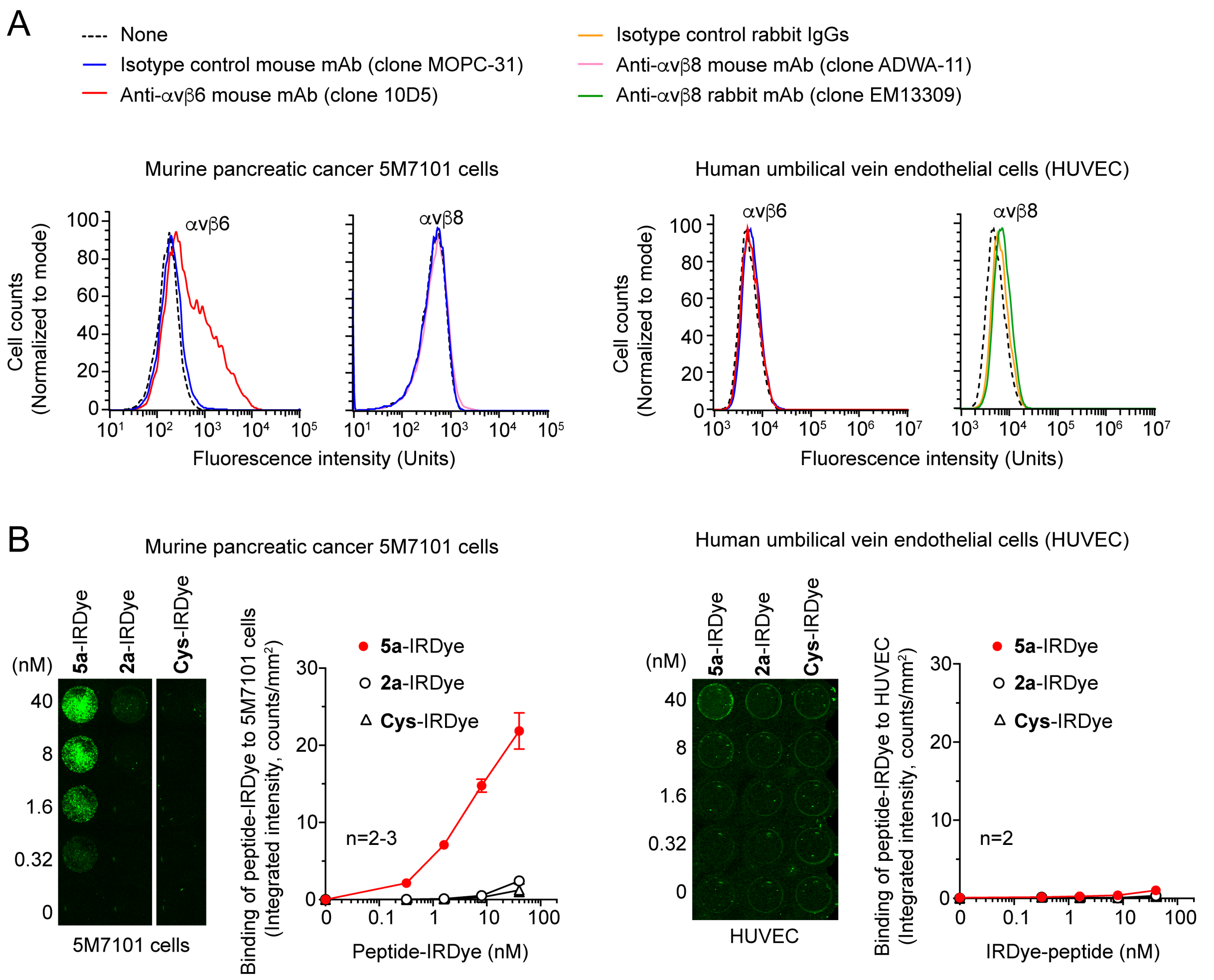

Figure S6. Binding of 5a-, 2a- and Cys-IRDye to 5M7101 and HUVEC cells.

A) Expression of αvβ6 and αvβ8 by 5M7101 and HUVEC cells assessed by FACS analysis using the indicated monoclonal antibodies, followed by an AlexaFluor 488-goat anti-mouse or anti-rabbit IgG polyclonal antibody.

B) Binding of the indicated peptide-IRDye conjugates to 5M7101 and HUVEC cells. Various amounts of the conjugates were added to cell monolayers (grown in 96-well microtiter plates) and incubated for 1 h at 37 °C, 5% CO_2. After washing, bound fluorescence was measured using an Odyssey CLx (LI-COR) scanner. Representative images of the scanned plates and quantification of the binding are shown. Mean ± SE of n=2-3 wells.

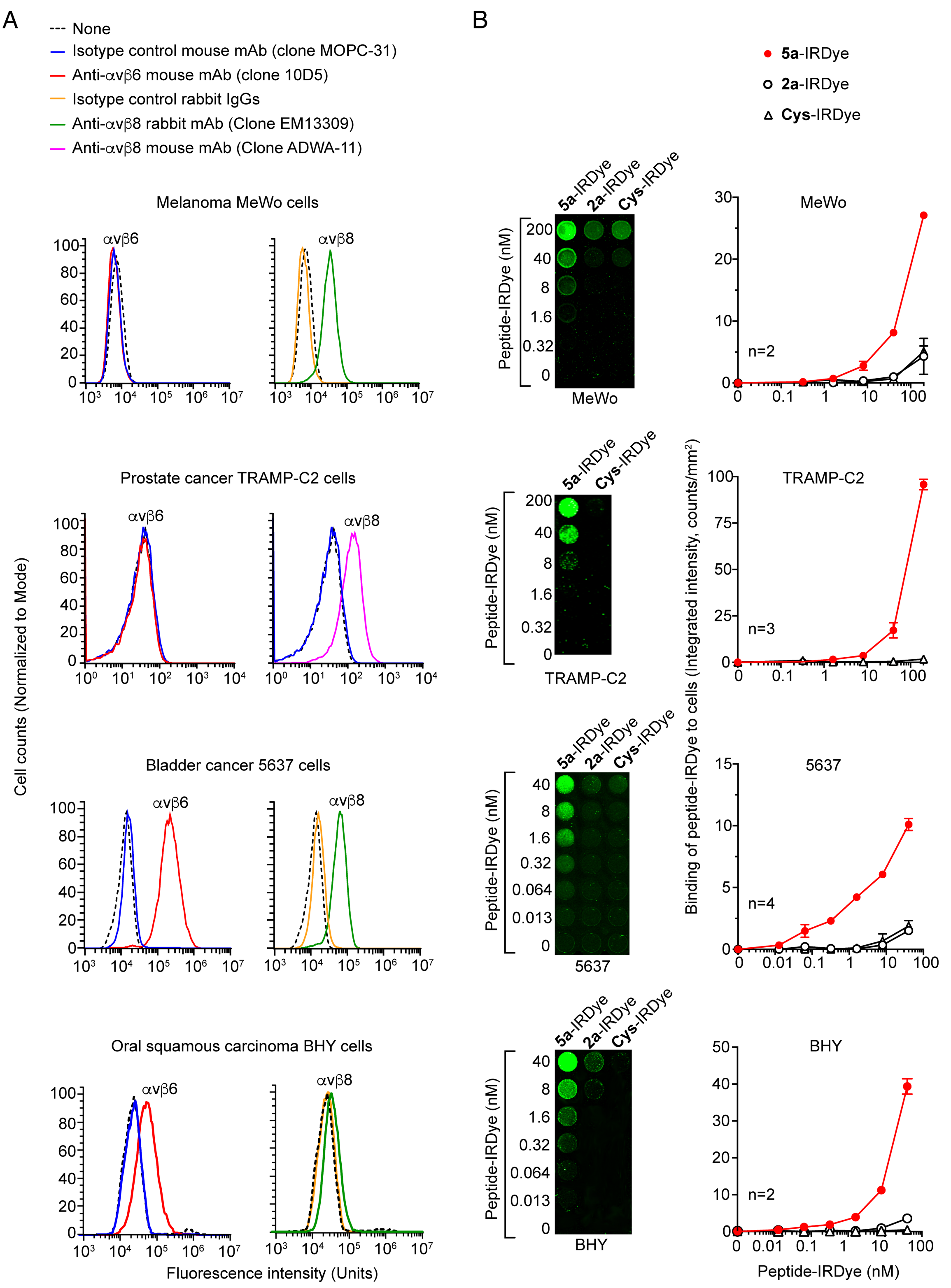

Figure S7. Binding of 5a-, 2a- and Cys-IRDye to MeWo, TRAMP-C2, 5637 and BHY and cells.

A) Expression of αvβ6 and αvβ8 by MeWo, TRAMP-C2, 5637 and BHY cells assessed by FACS analysis using the indicated monoclonal antibodies, followed by a AlexaFluor 488-goat anti-mouse or anti-rabbit IgG polyclonal antibody.
B) Binding of the indicated peptide-IRDye conjugates to MeWo, TRAMP-C2, 5637 and BHY cells. Various amounts of the conjugates were added to cell monolayers (grown in 96-well microtiter plates) and incubated for 1 h at 37 °C, 5% CO_2. After washing, bound fluorescence was measured using an Odyssey CLx (LI-COR) scanner. Representative images of the scanned plates and quantification of binding are shown. Mean ± SE of n=2-4 wells.

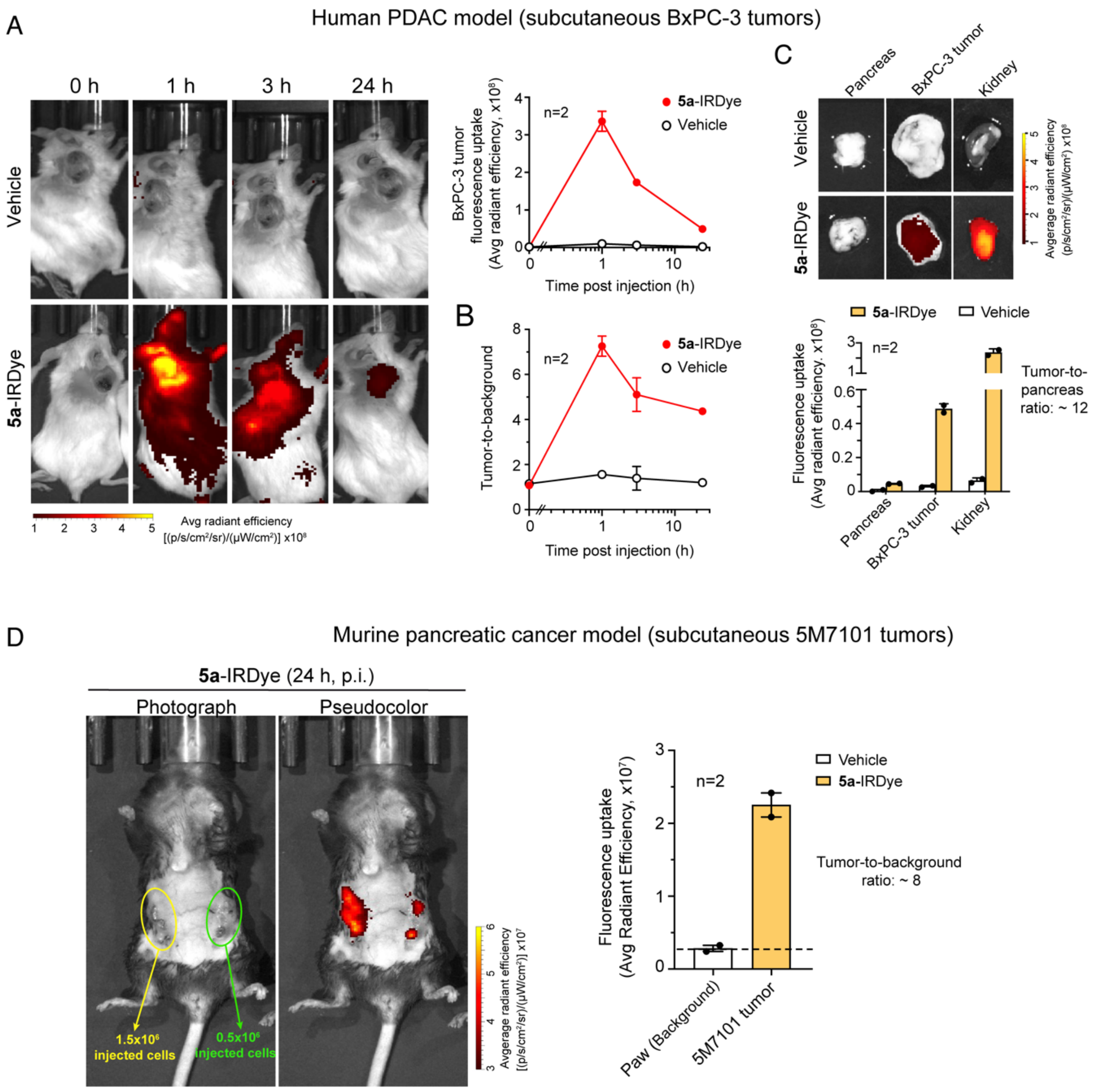

Figure S8. 5a-IRDye homes in on subcutaneous human and murine models of pancreatic cancer. A) Representative image and *in vivo* quantification of the **5a**-IRDye uptake in BxPC-3 tumors at 1, 3, and 24 h post-injection. NSG mice were challenged with BxPC-3 cells ($1x10^7$ cells) in the right shoulder. Thirty-five days later, mice were injected i.v. with 1.2 nmol of **5a**-IRDye or diluent (*vehicle*) and subjected to NIR-imaging using an IVIS imaging system. Mice treated with vehicle served as a reference for the quantification of autofluorescence in the NIR region. **B**) *Tumor-to-background ratio* over time. The fluorescence of forepaws was used to quantify the background. **C**) Representative *ex vivo* image and quantification of the fluorescence uptake in pancreas, BxPC-3 tumor, and kidney at 24 h post injection. **D**) Representative image and *in vivo* quantification of **5a**-IRDye uptake in 5M7101 tumors 24 h post-injection. C57BL/6N mice were challenged on the flanks with the indicated number of 5M7101 cells and 34 days later were treated i.v. with 1 nmol **5a**-IRDye. Graphs are means ± SE of 2 mice.

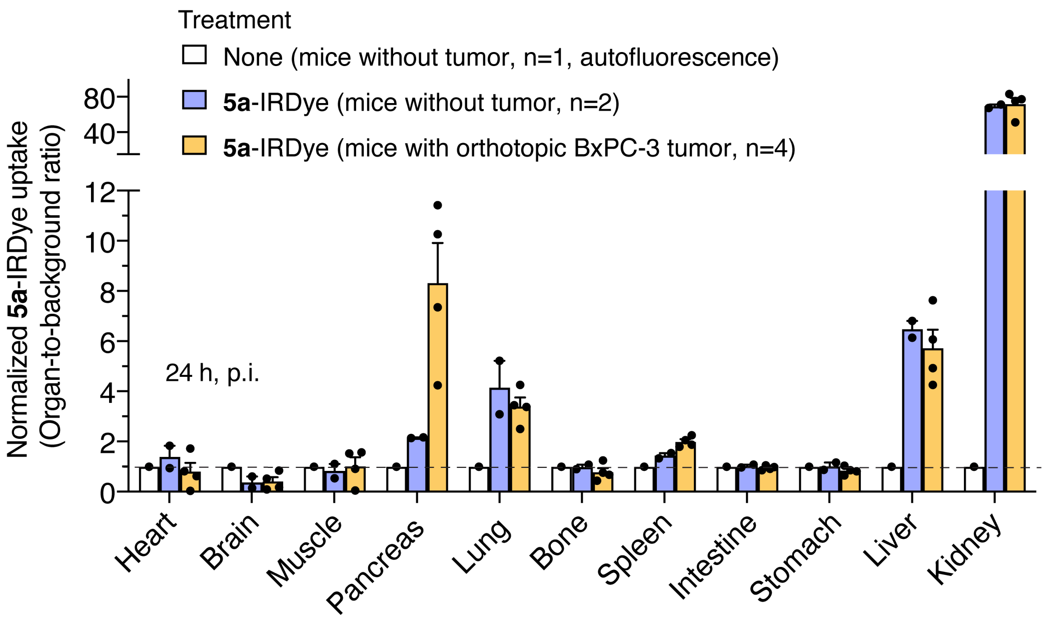

Figure S9. Normalized uptake of 5a-IRDye.

Data of **Figure 3C** have been normalized using the autofluorescence signal as background.

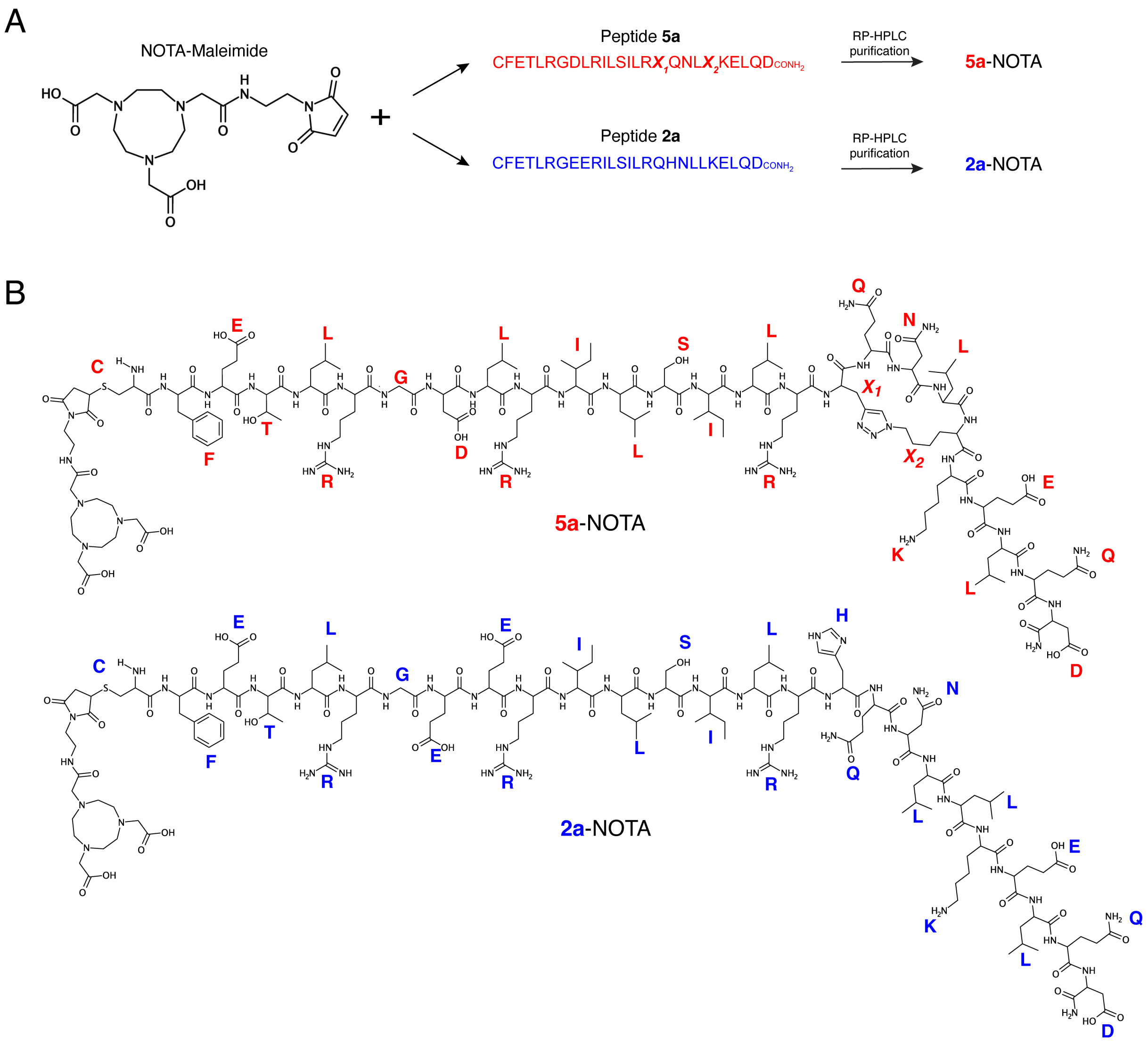

Scheme 2. Schematic representation of peptide-NOTA conjugate preparation (A) and conjugate structures (B).

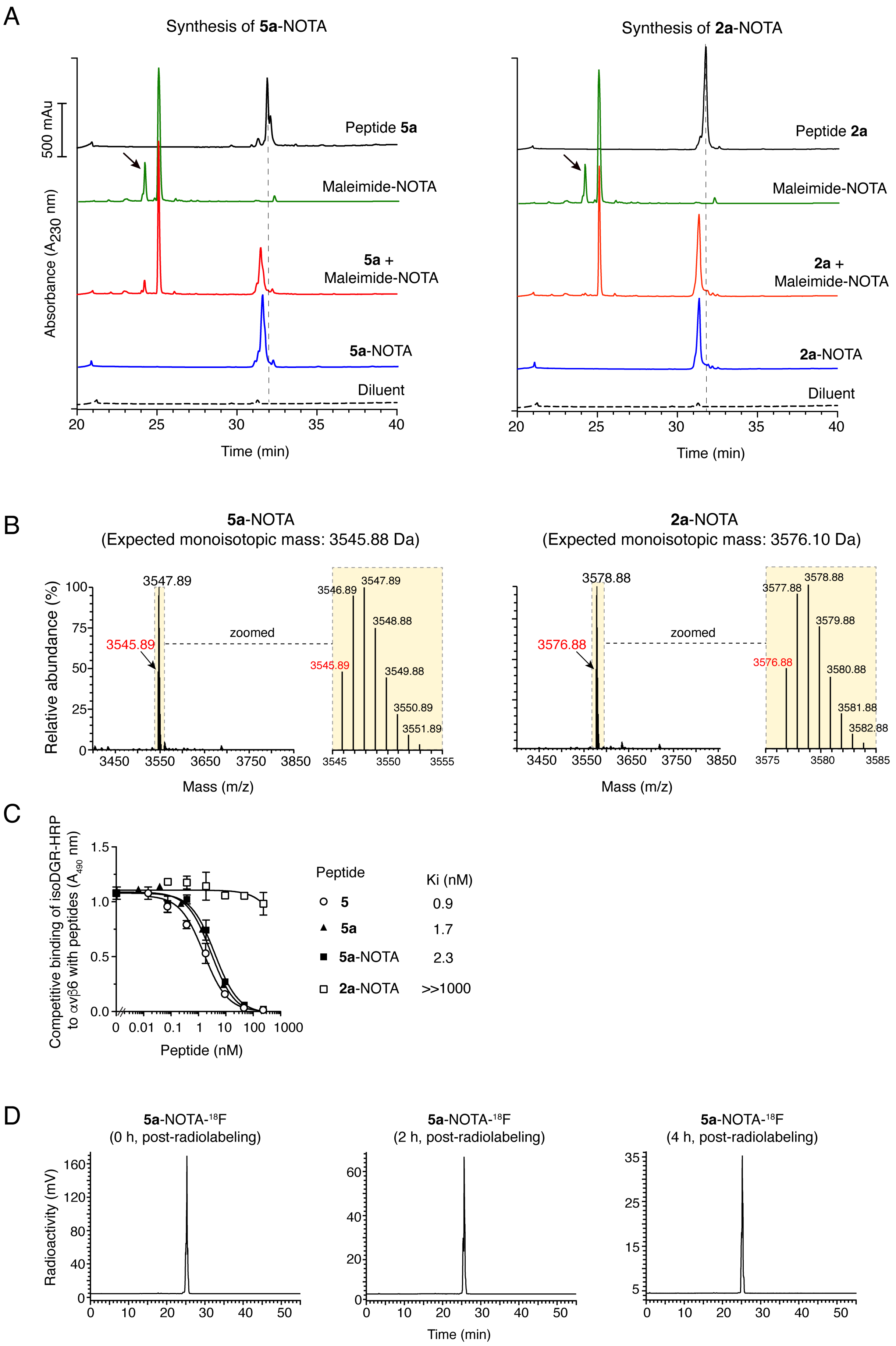
A
Synthesis of 5a-NOTA
Synthesis of 2a-NOTA
500 mAu
Absorbance (A230 nm)
Peptide 5a
Maleimide-NOTA
5a + Maleimide-NOTA
5a-NOTA
Diluent
Peptide 2a
2a + Maleimide-NOTA
2a-NOTA
Time (min)
B
5a-NOTA
(Expected monoisotopic mass: 3545.88 Da)
2a-NOTA
(Expected monoisotopic mass: 3576.10 Da)
Relative abundance (%)
3547.89
3545.89
zoomed
3546.89
3548.88
3549.88
3550.89
3551.89
3578.88
3576.88
3577.88
3579.88
3580.88
3581.88
3582.88
Mass (m/z)
C
Competitive binding of isoDGR-HRP to αvβ6 with peptides (A490 nm)
Peptide (nM)
Peptide
Ki (nM)
5 0.9
5a 1.7
5a-NOTA 2.3
2a-NOTA >>1000
D
5a-NOTA-18F (0 h, post-radiolabeling)
5a-NOTA-18F (2 h, post-radiolabeling)
5a-NOTA-18F (4 h, post-radiolabeling)
Radioactivity (mV)
Time (min)

Figure S10. Biochemical characterization of 5a- and 2a-NOTA conjugates.

A) RP-HPLC analysis of the single reagents (peptides and maleimide-NOTA) used for the preparation of conjugates, the products after 16 h of reaction (showing the unreacted materials and the products), and the final products after purification (**5a**- and **2a**-NOTA).

Peptide loaded on the column: 30-50 µg; *maleimide-NOTA*: 23 µg; *peptide-NOTA*: 40 µg. The arrows indicate the component of maleimide-NOTA "consumed" by the coupling reaction. Note that the final compounds (**5a**- and **2a**-NOTA) have a lower retention time (*dotted line*).

B) Mass spectrometry analysis (LTQ-XL Orbitrap) of purified **5a**- and **2a**-NOTA conjugates. The expected monoisotopic masses are shown. The arrows indicate the first peak of the monoisotopic distribution (which corresponds to the expected monoisotopic molecular weight), while the other peaks correspond to the same compound containing the heavier natural isotopes (mainly ^{13}C).

C) Competitive binding of *iso*DGR-HRP to αvβ6-coated microtiter plates with peptides and peptide-NOTA conjugates, as measured by competitive ELISA. A representative experiment is shown with the resulting *Ki* values. Mean ± SE of two technical replicates.

D) Stability of **5a**-NOTA after labeling with ^{18}F, as determined by RP-HPLC analysis using an ACE C18 column.

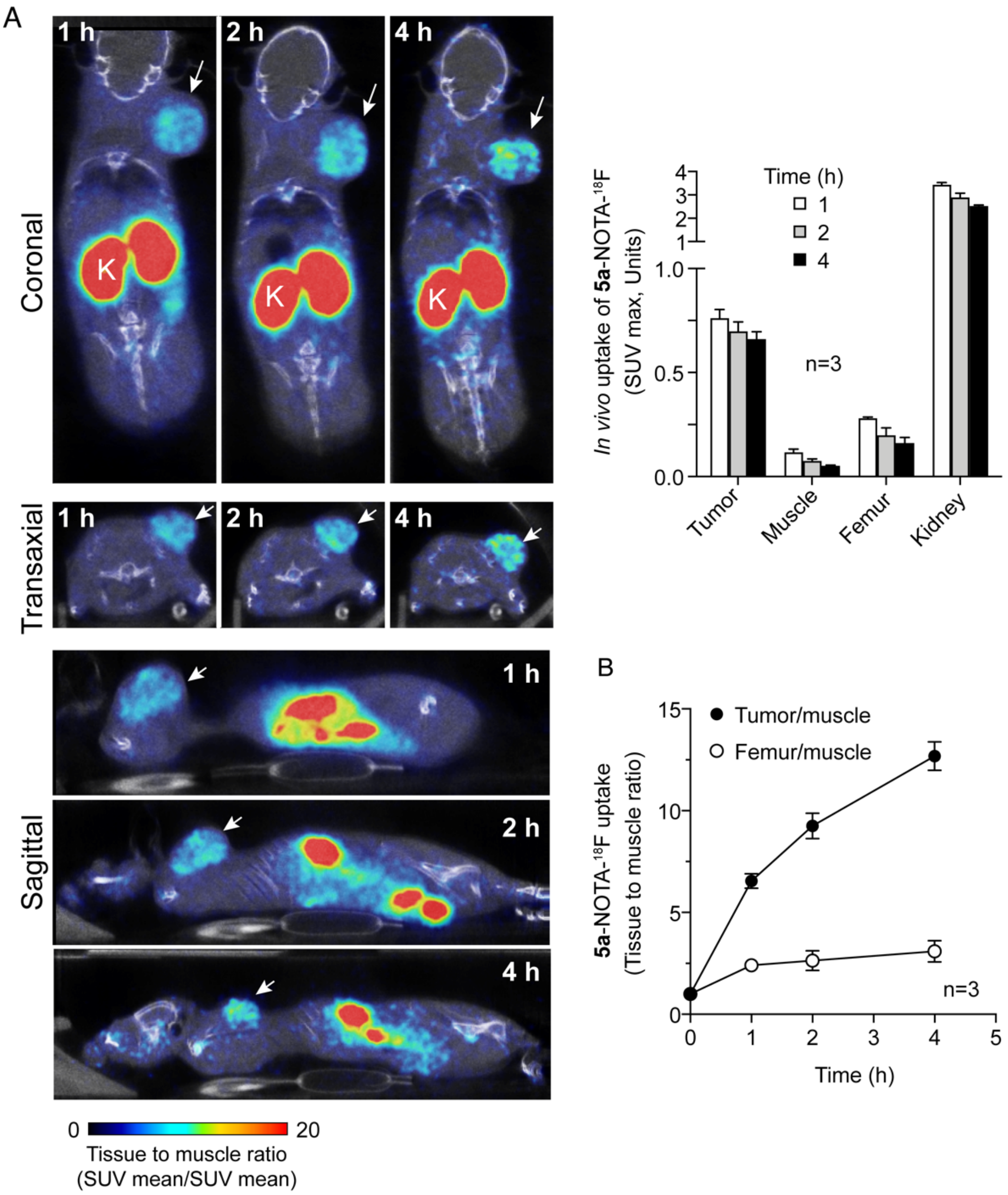

Figure S11. PET/CT assessment of 5a-NOTA-^{18}F uptake by subcutaneous BxPC-3 tumors.

NSG mice were challenged in the right shoulder with BxPC-3 cells and 30-35 days later were intravenously injected with **5a**-NOTA-^{18}F (~4 MBq/mouse) and subjected to whole-body PET/CT studies at the indicated time points.

A) A representative PET/CT image of a mouse for each time point (reported as tissue to muscle ratio of standardized uptake mean value, SUV mean) and the quantification of the standardized uptake maximum value (SUV max) of the radiotracer in the indicated tissues are shown. *Bars*, mean ± SE (n=3 mice). *Arrows*, BxPC-3 tumor.

B) Uptake of **5a**-NOTA-^{18}F in tumor or femur expressed as *tumor-* or *femur-to-muscle* ratio. Ratio values are presented as mean ± SE (n=3 mice).

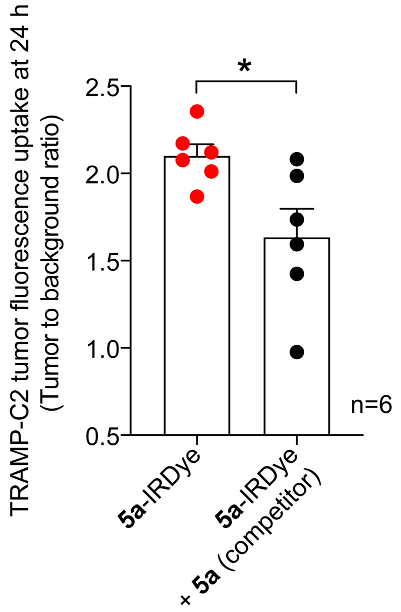

Figure S12. The uptake of 5a-IRDye by subcutaneous TRAMP-C2 tumors depends on the 5a moiety.

Immunodeficient mice were challenged in the right shoulder with TRAMP-C2 cells ($5x10^6$ cells/mouse) and injected i.v. 30-32 days later with or without an excess of unlabeled peptide **5a** (128 nmol/mouse). Two min later, mice were injected with **5a**-IRDye (1 nmol, i.v.), and fluorescence uptake in tumor was assessed after 24 h. Quantification of uptake of **5a**-IRDye in TRAMPC-2 tumors, expressed as the tumor-to-background ratio, is shown. Cumulative results of two independent experiments (*Bars*, mean ± SE, 2–4 mice/experiment).

*, P<0.05 by *two-tail t- test.*

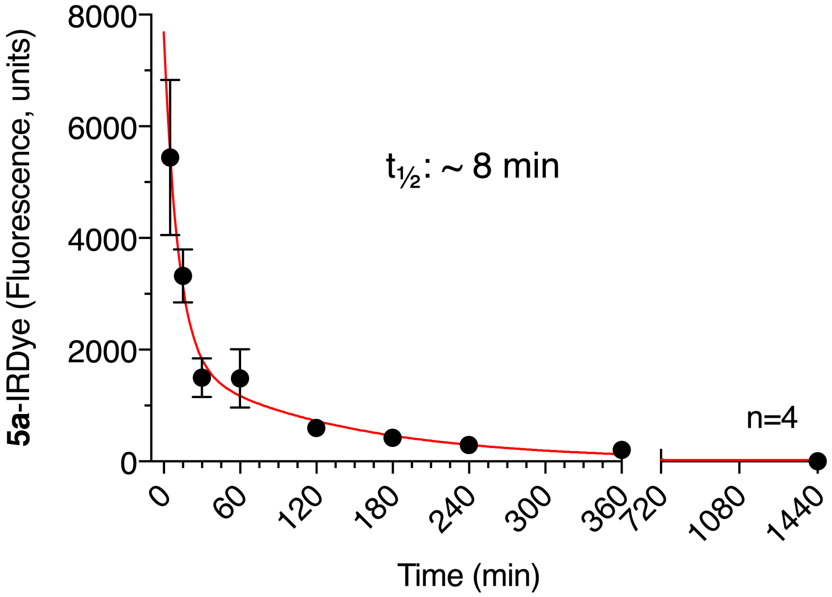

Figure S13. Plasma pharmacokinetics of 5a-IRDye in mice.

Eight BALB/c mice were divided in 2 groups (4 mice/group) and injected i.v. with **5a**-IRDye (1 nmol). Their blood was then collected into heparinized tubes at 5, 30, 120, 240, and 1440 min (group 1) or after 15, 60, 180, 360, and 1440 min (group 2). Plasma samples (20 µl) were diluted with PBS (50 µl, final volume) and transferred to a black 96-well microtiter plate. The amount of fluorescence in each sample was then quantified using an Infinite 200Pro reader plate (TECAN, excitation wavelength: 766 nm; emission wavelength: 810 nm; excitation bandwidth: 9 nm; emission bandwidth: 20 nm; gain: 180 optimal (100%) and number of flashes: 25). (Dots, mean ± SE, 4 mice). Plasma half-life of **5a**-IRdye was calculated using a two-phase exponential decay equation (GraphPad Prism software, San Diego California).

Supplemental References

1. Dodagatta-Marri E, Ma HY, Liang B, Li J, Meyer DS, Chen SY, et al. Integrin alphavbeta8 on T cells suppresses anti-tumor immunity in multiple models and is a promising target for tumor immunotherapy. Cell Rep. 2021; 36: 109309.
2. Curnis F, Gasparri AM, Longhi R, Colombo B, D'Alessio S, Pastorino F, et al. Chromogranin A binds to avb6-integrin and promotes wound healing in mice. Cell Mol Life Sci. 2012; 69: 2791-803.
3. Nardelli F, Ghitti M, Quilici G, Gori A, Luo Q, Berardi A, et al. A stapled chromogranin A-derived peptide is a potent dual ligand for integrins alphavbeta6 and alphavbeta8. Chem Commun (Camb). 2019; 55: 14777-80.
4. Kim MP, Evans DB, Wang H, Abbruzzese JL, Fleming JB, Gallick GE. Generation of orthotopic and heterotopic human pancreatic cancer xenografts in immunodeficient mice. Nat Protoc. 2009; 4: 1670-80.
